# Revealing hidden defects through stored energy measurements of radiation damage

**DOI:** 10.1126/sciadv.abn2733

**Published:** 2022-08-03

**Authors:** Charles A. Hirst, Fredric Granberg, Boopathy Kombaiah, Penghui Cao, Scott Middlemas, R. Scott Kemp, Ju Li, Kai Nordlund, Michael P. Short

**Affiliations:** ^1^ Department of Nuclear Science and Engineering, Massachusetts Institute of Technology, Cambridge, MA 02139, USA.; ^2^ Department of Physics, University of Helsinki, P.O. Box 43, FIN-00014 Helsinki, Finland.; ^3^ Materials and Fuels Complex, Idaho National Laboratory, Idaho, ID 83415, USA.; ^4^ Department of Mechanical and Aerospace Engineering, University of California, Irvine, Irvine, CA 92697, USA.; ^5^ Department of Materials Science and Engineering, Massachusetts Institute of Technology, Cambridge, MA 02139, USA.

## Abstract

With full knowledge of a material’s atomistic structure, it is possible to predict any macroscopic property of interest. In practice, this is hindered by limitations of the chosen characterization techniques. For example, electron microscopy is unable to detect the smallest and most numerous defects in irradiated materials. Instead of spatial characterization, we propose to detect and quantify defects through their excess energy. Differential scanning calorimetry of irradiated Ti measures defect densities five times greater than those determined using transmission electron microscopy. Our experiments also reveal two energetically distinct processes where the established annealing model predicts one. Molecular dynamics simulations discover the defects responsible and inform a new mechanism for the recovery of irradiation-induced defects. The combination of annealing experiments and simulations can reveal defects hidden to other characterization techniques and has the potential to uncover new mechanisms behind the evolution of defects in materials.

## Teaser

The combination of annealing experiments and simulations can uncover new defect evolution mechanisms in materials.

## INTRODUCTION

At the most fundamental level, a material’s properties are determined by its structure. Thus, with full knowledge of the structure, it is possible to predict a material’s behavior. In practice, this is limited by the (in)ability of a given characterization technique to resolve the full structure, especially at the atomic level. This general problem is exemplified by the study of irradiation-induced defects in materials.

Irradiation alters materials through the creation of defects (*1*). To predict how the properties will change, it is critical to characterize the type, size, and number density of these defects. Despite many techniques being used—transmission electron microscopy (TEM), positron annihilation spectroscopy (PAS), and resistivity measurements—each of these methods has limitations that restrict their ability to fully characterize the defects in irradiated materials. Simulations and experiments show that the majority of defect clusters are below ∼10s of point defects in size and are thus below the resolution limit for TEM (*2*, *3*). Consequently, TEM often underestimates the defect density by an order of magnitude (*4*, *5*). PAS can detect individual vacancies but is not sensitive to interstitials (*6*), which prevents the characterization of a substantial fraction of defects. Resistivity measurements have been extensively used but require knowledge of the resistivity contributions from each defect type to interpret the microstructure (*7*). This is complex and is computationally intractable to be simulated for systems larger than ∼100s of atoms (*8*). Instead of determining a material’s structure through spatial characterization or property measurement, it may be more effective to probe another dimension: the energy space.

By definition, defects are imperfections within a crystal, and therefore, all defects have an associated excess energy. In addition, the evolution of defects is limited by an energy barrier. Thus, for every defect reaction, there is a characteristic activation energy and energy of transformation. Determination of these parameters, through kinetic methods such as Kissinger analysis (*9*), allows the defects involved to be deduced. While energetic transitions in more complex material systems may overlap, the deconvolution of simultaneously evolving microstructural features can be aided by correlative techniques.

That energy can be stored in a material, in the form of irradiation-induced defects, was first postulated by Eugene Wigner during the Manhattan Project (*10*). Since then, there have been many studies investigating Wigner energy in ceramic materials, including graphite (*11*, *12*), Si (*13*), and SiC (*14*). Metals have been less well studied, with most analyses focused on defect annealing after cryogenic irradiation. These include experiments on Cu (*15*), Al (*16*), Be (*17*), and Mg (*18*). However, evaluating defect populations through annealing experiments need not be limited to cryogenic temperatures. This concept is applicable to defects at all temperatures.

Exploring defects through their energetic dimensions allows the direct comparison between experimental annealing and molecular dynamics (MD) simulations of defect evolution (*19*). This combination of techniques can reveal defects that are hidden to other characterization techniques and thus has the potential to uncover new mechanisms behind the evolution of defects. Experimental and simulated annealing is not limited to characterizing defects arising from irradiation but can be used to investigate damage resulting from other environmental factors experienced in the processing and operation of materials. In addition, this approach can be applied to study defects across the whole range of materials systems: from structural to optical to electronic materials.

## RESULTS

Here, we demonstrate the excess energy idea by conducting differential scanning calorimetry (DSC) experiments to anneal neutron-irradiated Ti and determine the stored energy corresponding to radiation damage recovery. Multiple energy release stages were observed between 300° and 600°C, which contrasts with the established recovery model. TEM provides some insight into the defects responsible but cannot fully account for the stored energy released. Experiments are correlated to MD simulations of radiation damage annealing to investigate the defects involved in the recovery mechanism. Comparisons between the experimental and simulated results support the use of stored energy to explore defects which may not be measurable by other characterization techniques.

### DSC annealing experiments

**Fig. 1.**
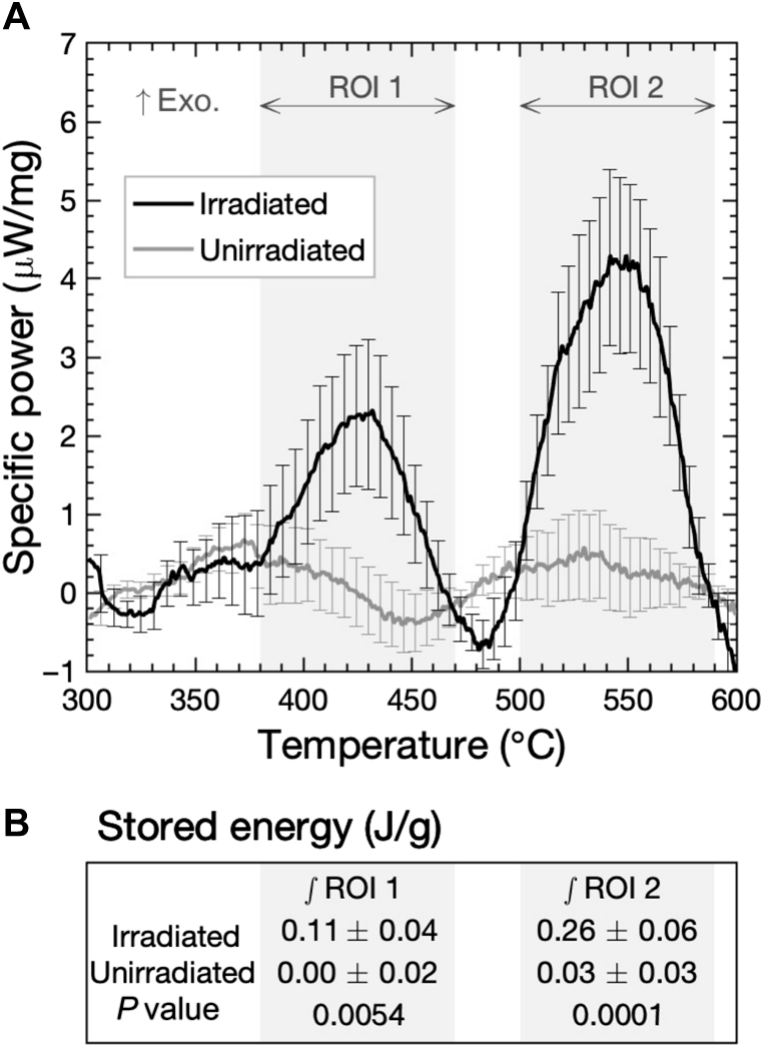
Irradiated samples release stored energy during annealing. (**A**) Curves show the specific power difference between the first heat of the (defected) sample and the mean of subsequent heats 2 to 5 (annealed sample). Each dataset is the mean of nine samples, and the error bars show **±** the summation in quadrature of the SEs arising from averaging multiple corrections, heats, and samples. (**B**) Integrating the stored energy within each ROI shows that irradiated samples yield statistically significant results. Uncertainties are calculated as the summation in quadrature of the SEs arising from averaging the integrals of sample and correction runs. The full data analysis procedure is described in section S2.1.

Figure 1 shows the difference in specific power, between the first heat of the (defective) sample and the mean of subsequent heats 2 to 5 (annealed sample), for irradiated and unirradiated samples. Significantly, the irradiated samples exhibit exothermic peaks, whereas the unirradiated samples do not. It is also notable that there are two distinct peaks observed. The irradiated samples exhibit exothermic energy releases between 380° and 470°C [region of interest (ROI) 1] and 500° and 590°C (ROI 2). The presence of multiple peaks indicates that two separate annealing processes occur between 300° and 600°C. This contrasts with the established recovery model (*20*), which describes one process occurring during stage V, and implies that the process of recovery is more complex than previously thought.

Integrating the signal within each ROI yields values for the stored energy (in J/g). ROI 1 corresponds to a release of 0.11 ± 0.04 J/g in the irradiated samples, compared to 0.00 ± 0.02 J/g in the unirradiated samples. ROI 2 corresponds to 0.26 ± 0.06 and 0.03 ± 0.03 J/g for irradiated and unirradiated samples, respectively. These measured values of stored energy are the correct order of magnitude for radiation damage in metals (*14*) and show statistical significance between the irradiated and unirradiated samples. To explore the defect reactions behind each of the peaks, we conducted TEM to visualize the evolution of extended defects.

### TEM characterization

**Fig. 2.**
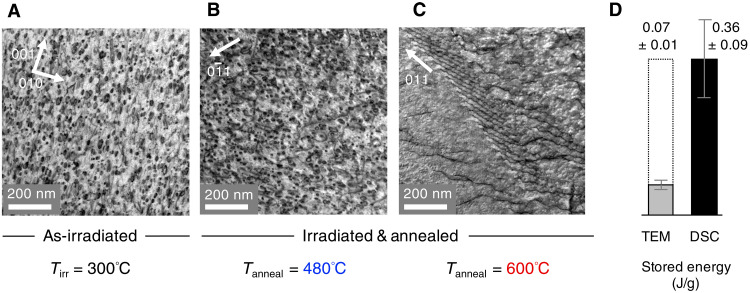
TEM provides insight into the annealing mechanism but cannot fully account for the energy released. (**A**) The as-irradiated microstructure shows a high density of ***<*a*>***-type dislocation loops. The scanning TEM (STEM) image was taken by tilting the TEM specimen to the two-beam diffraction condition of **g** = 011 along the zone axis of [100]. (**B**) Following annealing to 480°C, the microstructure has changed very little. The STEM image was taken with the two-beam diffraction condition of $g=0\bar{1}1$ along the [311] zone axis. (**C**) After heating to 600°C, the microstructure has recovered. The STEM image was taken with $g=01\bar{1}$ along the [111] zone axis. (**D**) Calculating the stored energy contribution from the dislocation loops shows that the TEM-visible defects represent only a fraction of the energy measured in the DSC between 300° and 600°C. The error bars show the SE of the stored energy integrated between 380° and 590°C for each sample (DSC) and the SD of the energy calculation. Full details of the calculation are shown in section S4.

Figure 2 shows micrographs of the irradiated and annealed samples. In the as-irradiated sample, there is a high density of <**a**>-type dislocation loops. This qualitatively matches that reported previously for Ti irradiated to a 3× greater fluence (1.7 × 10^25^ m^−2^) at a similar temperature (316°C) (*21*). The mean dislocation loop diameter in our samples is 19 nm, and the number density is (4.0 ± 0.7) ×10^21^ m^−3^. Following annealing to 480°C, the microstructure is remarkably similar. The mean dislocation loop diameter is still 19 nm, and the number density is unchanged at (4.0 ± 0.8) ×10^21^ m^−3^. After heating to 600°C, the dislocation loops have disappeared and the microstructure has fully recovered, showing features that are characteristic of annealed metals.

Calculating the energy per dislocation loop (details in section S4) allows the stored energy contribution from TEM-visible defects to be compared to the DSC measurements. Figure 2D shows that TEM-visible dislocation loops in the as-irradiated sample contribute a stored energy density of 0.07 ± 0.01 J/g compared to our DSC measurements of 0.36 ± 0.09 J/g (released between 300° and 600°C). This is notable, as it demonstrates that TEM-visible defects cannot fully account for the stored energy release, and implies that there are defects being annealed, which are not detected by the TEM. This finding is consistent with the well-known fact that TEM cannot resolve the full spectrum of defects (*3*, *22*) in the material and supports the use of DSC measurements to quantify the magnitude of this discrepancy. To investigate the annealing mechanism and explore the nature of the “hidden” defects, we conduct MD simulations.

### MD annealing simulations

**Fig. 3.**
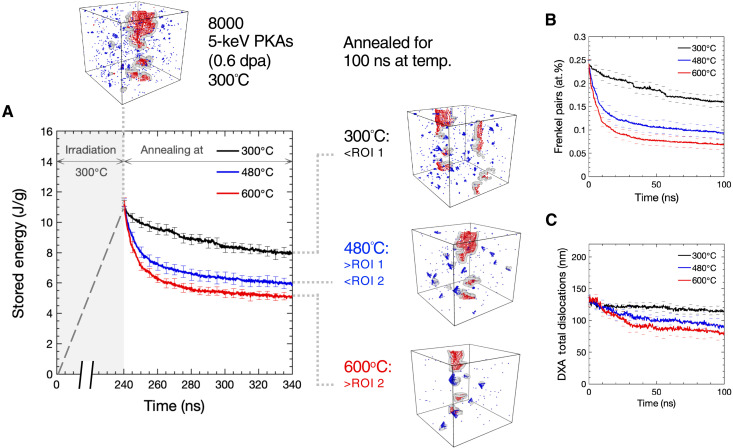
Simulated annealing of radiation damage shows that the stored energy recovery has a notable contribution from point defects. Simulations of 8000 primary knock-on atom (PKA) cascades generate defected microstructures. These are then annealed for 100 ns at 300°, 480°, or 600°C to determine the energy release (**A**) and the defects remaining. WS and DXA analyses show the defects as a function of time, with (**B**) a large decrease in the point defect concentration and (**C**) a minimal decrease in the total dislocation length. All data shown are means of 10 independent simulations, and the errors bars are **±**SE. dpa, displacements per atom; at.%, atomic %.

Figure 3 shows the evolution of irradiation-induced defects during annealing. Initially, the microstructure consists of isolated vacancies, small vacancy clusters, and <**a**>-type interstitial dislocation loops. A representative atomic configuration is shown with dislocations represented as gray lines, interstitials as red spheres, and vacancies as blue spheres. Simulation cells were annealed for 100 ns at 300°, 480°, or 600°C, and the corresponding stored energy is plotted as a function of time in Fig. 3A. For all temperatures, initially, the stored energy decreases rapidly, and then the rate diminishes until it effectively plateaus toward 100 ns. Annealing at 300°C exhibits stepwise behavior with periods of gradual recovery between larger drops in stored energy.

Figure 3 (B and C) shows the corresponding Wigner-Seitz (WS) and dislocation extraction algorithm (DXA) analyses as measures of the point defect and dislocation populations, respectively. It can be seen that the point defect evolution closely matches the stored energy behavior, while the total dislocation line length is either constant (300°C) or decreases slightly (480° and 600°C).

**Fig. 4.**
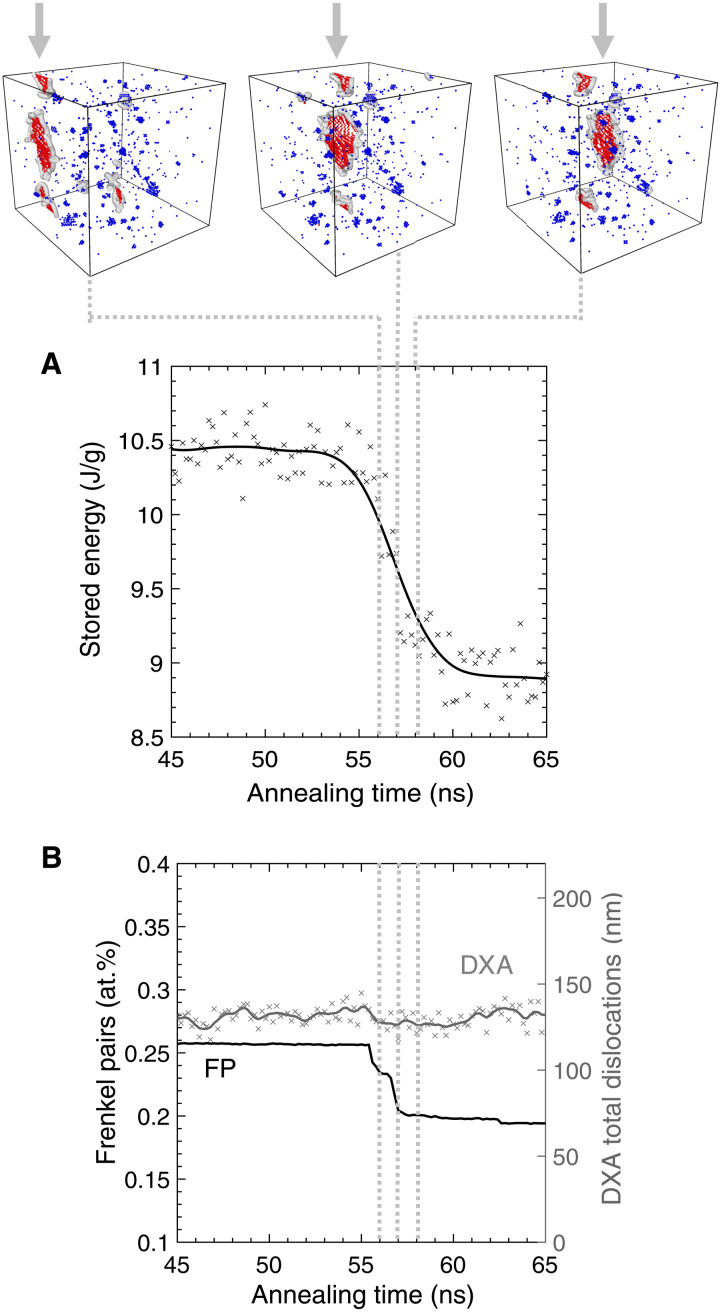
Stored energy recovery occurs via dislocation loops gliding and annihilating point defects. (**A**) The stored energy of the system during relaxation at 300°C. Atomic configurations (above) highlight the associated migration of a dislocation loop. (**B**) WS and DXA analyses show that during this process, the number of point defects decreases, while the total line length of dislocations does not change.

Figure 4 investigates the mechanism behind the stored energy evolution in detail. During the notable decrease in stored energy, dislocation loops glide considerable distances (>20 nm) and annihilate vacancies during this process. Figure 4B shows that the motion of dislocation loops is associated with a large decrease in point defect concentration, while the total dislocation line length remains constant. This process is consistent with our experimental results that show that ROI 1 involves an exothermic defect reaction but yields microstructures that appear similar when evaluated using TEM. The annihilation of vacancies by the interstitial-type dislocation loop should ultimately lead to a decrease in the dislocation loop size. The discrepancy between dislocation line length and point defect concentration may result from a delay in the reorganization of the dislocation loop.

The glide of loops and recombination of vacancies is responsible for the considerable recovery seen initially at all temperatures and periodically in the 300°C anneal. After the dislocation loops glide through the supercell, their migration is reduced. As the simulation temperature is constant throughout, it can be postulated that the driving force for glide comes from the stress fields of defects interacting, and when the small defect clusters have been annihilated, there is no longer a sufficient driving force for migration. The exhaustion of point defects may be an artifact of the limited simulation cell size, and in a more realistic microstructure, the dislocation loops may glide until sinking at a grain boundary or interacting with another dislocation.

## DISCUSSION

The evolution of irradiation-induced defects is more complex than previously thought. This may be due to previous studies being limited by the characterization techniques used. Exploring defects through their energetic dimensions can yield insight into their formation and evolution, as demonstrated by our work.

### Combined annealing experiments and simulations infer defect evolution

DSC measurements have been used to quantify the stored energy release from radiation damage annealing in metals. The observed temperature range of recovery matches that observed for hardness recovery of fast neutron-irradiated Ti (*23*). Notably, our experiments show two distinct peaks corresponding to two separate processes where the established recovery model predicts only one (*20*). The temperature range of ROI 1 matches a prior PAS annealing study of neutron-irradiated Ti (*24*), and the ROI 2 temperature range corresponds to recovery of cold-worked Ti (*25*, *26*). This suggests that the two stages involve vacancies and dislocations, respectively.

TEM characterization supports these findings. The high density of dislocation loops observed in the as-irradiated sample remains after annealing to 480°C. Following annealing to 600°C, the dislocation loops recover. Comparing the DSC and TEM results, by converting the TEM-measured defect density to a stored energy density, shows that TEM-visible defects make up only a fraction of the energy released. This indicates that there are defects involved in the annealing process that are below the resolution of the TEM.

MD simulations of primary knock-on atom (PKA) cascades generate microstructures that are qualitatively similar to the established recovery model (*20*) and prior TEM results. Interstitial dislocation loops and smaller vacancy clusters match that expected for temperatures above stage III recovery, and <**a**>-type interstitial dislocation loops have been observed previously in the TEM (*21*). While the simulations do not contain vacancy dislocation loops or network dislocations, this may be explained by the high effective dose rate. The elevated dose rate results in greater recombination of defects and, thus, less growth of vacancy clusters into dislocation loops and less coalescence of interstitial dislocation loops into network dislocations. In addition, since the simulations are only (∼20 nm)^3^ in volume, with periodic boundary conditions, extended dislocations are unlikely to form. While the presence of existing dislocations and grain boundaries would influence the evolution of radiation damage, not including them in our simulations is motivated by the difference in length scales between damage production and existing microstructure. The areal density of defect clusters in our experimental samples is much larger (2.5 × 10^14^ m^−2^) than that of network dislocations (2.4 × 10^13^ m^−2^) and is also considerably larger (by many orders of magnitude) than that of grain boundaries. Thus, the shortest defect-defect distance is between point defect clusters and the high density of dislocation loops. As a result, this will be the most pertinent interaction, both in terms of the reaction rate and also in terms of the stored energy density.

Analyzing the defect annealing simulations shows a strong correlation between the stored energy and the Frenkel pair concentration within the system. Investigating the mechanism behind the stored energy recovery reveals that dislocation loops glide through a field of point defects annihilating them. Previous in situ TEM heating experiments (*27*) of proton-irradiated Zr observed gliding of <**a**> loops between 300° and 425°C. The considerable decrease in system energy driven by point-defect induced migration of dislocations has also been observed in simulations by Derlet and Dudarev (*28*). The observed mechanism is similar to the effect of dislocation channeling in a highly damaged metal. In that process, dislocations become mobile and sweep straight regions of material free of smaller dislocations, creating a defect-free “channel” (*29*, *30*). However, the current mechanism is clearly distinct from this since the dislocations are much smaller, the damage level at which the effect occurs is lower, and the defects cleared away are point defects and small defect clusters.

### A new mechanism for elevated-temperature irradiation damage recovery

**Fig. 5.**
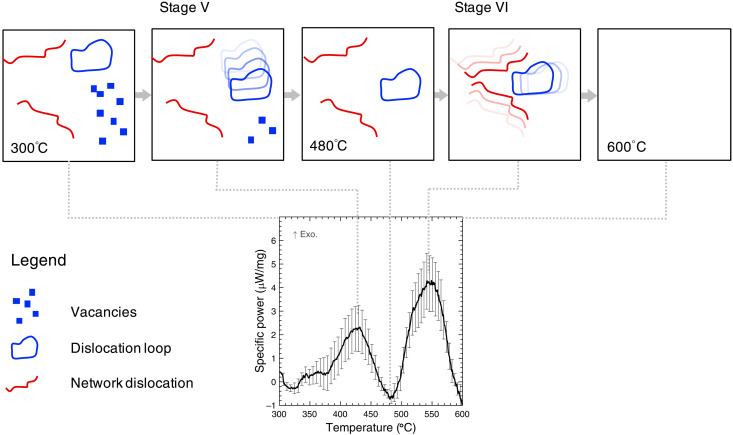
Recovery of neutron-irradiated Ti consists of two stages: Dislocation loop glide followed by dislocation recovery. DSC, TEM, and MD annealing results suggest that between 300° and 600°C, there are multiple recovery stages. Stage V: Glide of dislocation loops through a field of point defects and small clusters, annihilating them. Stage VI: Recovery of dislocation loops and network dislocations.

Interpreting all our results leads to the following proposed mechanism for recovery, as seen in Fig. 5. Initially, the irradiated microstructure consists of isolated vacancies and small vacancy clusters, dislocation loops, and network dislocations that form because of the impingement of dislocation loops. Heating between 300° and 480°C leads to stage V: Dislocation loops become mobile and glide through the system sweeping up vacancies. Heating between 480° and 600°C leads to stage VI: Dislocation loops and network dislocations become mobile and annihilate leading to an annealed microstructure.

This mechanism contrasts with the established recovery model (*20*) in a number of ways. First, we observe two distinct processes at temperatures corresponding to (so-called) stage V where the model predicts only one. Given that multiple substages exist for earlier recovery stages (*31*), this finding is not completely unexpected. Stages V and VI have also previously been reported for neutron-irradiated W, with the authors attributing them to the annealing of vacancies and the recovery of complex defects such as dislocation loops, respectively (*32*). This prior work agrees well with our postulated mechanism. Second, the glide of dislocation loops also does not feature in the recovery model, which assumes the evaporation of point vacancies from sessile vacancy defect clusters and their annihilation at larger interstitial clusters. In addition, the coalescence of interstitial loops into network dislocations is not captured by the model. This may be due to the irradiation conditions (cryogenic and electron) and characterization techniques (resistivity) used for many of the prior studies. Electron irradiation creates isolated Frenkel pairs within the material. Upon heating through stages I to IV, many defects will have recombined, and the remaining dislocation loops may not be large enough to coalesce and form network dislocations. Last, resistivity is less sensitive to network dislocations than to small defect clusters, and the resistivity values may well have recovered close to the preirradiation value. Our work highlights the importance of understanding the conditions in which previous mechanisms have been discovered and the limitations of their extrapolation to different scenarios. As a result, our proposed mechanism may be more applicable to practical investigations of radiation damage at reactor-relevant temperatures.

To conclusively determine this mechanism, additional characterization is being conducted. X-ray diffraction will be used to determine the defect densities before and after annealing at 480° and 600°C and thus deduce the change in defect populations. This will be supported by PAS measurements to validate the change in vacancy concentration with temperature. In addition, in situ TEM heating will be used to observe the evolution of larger defects directly. Further DSC experiments, at different heating rates, will be conducted to determine the activation energy for each of the annealing peaks. These can be correlated to nudged elastic band simulations to determine the activation energy of loop migration with and without point defects. Simulations with larger supercell sizes may enable the study of loop coalescence into extended defects, exploring the dislocation recovery; however, computational cost may be a limiting factor. Note also that the MD annealing simulations are likely only representative of the first annealing peak observed in the DSC (ROI 1), as there are no network dislocations or grain boundaries that would enable the sinking of dislocation loops as predicted in our mechanism for the second annealing peak (ROI 2). While there is a difference in composition between our experimental samples, which are commercially pure Ti, and our simulations, which are completely pure Ti, solute atoms may be trapped at point vacancies and their clusters rather than at dislocation lines. Atom probe tomography is being conducted to confirm the location of solutes and thus determine their effect on our proposed recovery mechanism.

### Microstructural understanding is only as good as our characterization techniques

Our DSC experiments show that two energetically distinct processes occur in place of stage V recovery, and consequently, the annealing mechanism for irradiation-induced defects is more complex than previously thought. This is supported by the paper of Blewitt *et al*. (*33*), which shows a discrepancy between resistivity and yield stress recovery of neutron-irradiated Cu (*33*). This implies that different populations of defects present in the material are responsible for the resistivity and yield stress and demonstrates the perils of using certain characterization techniques to investigate the microstructure of a material. Dennett *et al*. (*34*) recently demonstrate that “the evolution in elastic properties during swelling is found to depend significantly on the entire size spectrum of defects, from the nano- to meso-scales, some of which are not resolvable in imaging.” Limitations on the sensitivity of characterization techniques, such as electron microscopy, restrict analysis to a subset of the defects present and may result in the development of inaccurate models. As a result, using these techniques to correlate the structure of a defected material to its behavior will be unsuccessful (*4*, *5*).

Instead of spatial characterization, defects can be identified and quantified through their excess energy. Fundamentally, all defects in a material contribute to its energetic structure, and therefore, all defects have the ability to be detected through changes in their population. Crucially, annealing experiments can be directly compared to MD simulations to gain insight into the defect reactions occurring. This also has the potential to experimentally validate atomistic simulations, thereby answering the key question that exists for all simulated observations. In conclusion, exploring microstructure through the lens of stored energy can be applied to the whole spectrum of material systems, can reveal defects unable to be detected by other characterization techniques, and has the potential to uncover new mechanisms behind the evolution of defects.

## MATERIALS AND METHODS

### Materials

Samples were sectioned from a one-half inch CP-2 titanium nut. The composition is given in Table 1. The nut was subject to 73 days of irradiation in the Advanced Cladding Irradiation (ACI) facility of the Massachusetts Institute of Technology reactor. The conditions in the ACI loop simulate a pressurized water reactor with controlled coolant chemistry and temperature of 300° ± 2°C. The nut was irradiated at a fast neutron flux of 1.0 × 10^14^ cm^−2^ s^−1^ (>0.1 MeV) to a total fluence of 6.3 × 10^20^ cm^−2^. This corresponds to a dose of 0.76 displacements per atom (dpa), which was calculated using the Norgett-Robinson-Torrens formula (*35*) and the total damage energy production cross section (ENDF/B-VIII.0 database, MT=444 cross section) with *E*_d_ = 30 eV (*36*).

**Table 1.** Composition of CP-2 Ti hex nut. [O, N] was determined using inert gas fusion; [C] was determined using combustion infrared detection, and all other elements were determined using direct current plasma emission spectroscopy. wt %, weight %.

	**Ti**	**O**	**Fe**	**N**	**C**	**Al**	**Ni**	**Mn**	**Si**	**Sn**
wt %	balance	0.117	0.081	0.029	0.028	0.013	0.013	0.011	0.0073	0.0070

After irradiation, the nut was sectioned on a low-speed saw into approximately (4 mm)^3^ samples, with the average mass of 67 mg for DSC analysis. While mechanical deformation induces cold work to the sectioned faces, this contributes negligibly to the stored energy because of the surface area to volume ratio of the samples. In addition, this contribution will be identical for unirradiated and irradiated samples and can thus be accounted for.

Unirradiated samples were sectioned from an identical nut; a subset of these were annealed in a vacuum furnace at 300°C for 16 hours or at 400°C for 168 hours to replicate the time spent at 300°C in the reactor. Unirradiated samples show no measurable difference in stored energy with prior annealing; they show no measurable release of stored energy between 50° and 600°C.

### DSC experiments

**Fig. 6.**
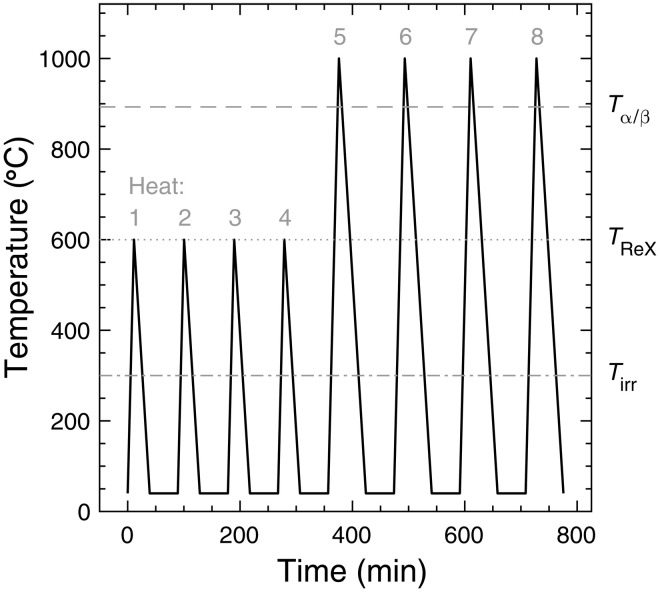
DSC heating profile. Samples were heated initially (heats 1 to 4) to anneal out radiation damage and generate an annealed baseline and then were heated (heats 5 to 8) through the α/β phase transition to measure the enthalpy of transformation. *T*_ReX_ = recrystallization temperature.

Samples were annealed using a NETZSCH 404 F3 DSC with a type P sensor for increased sensitivity. Crucibles were 0.19~ml Pt/Rh to maximize the sample size, and Y_2_O_3_ spray was used to ensure that samples did not adhere to the crucibles. Samples were heated at 50 K/min in an ultra high purity Ar atmosphere, according to the heating profile shown in Fig. 6. Samples were heated to 600°C four times, first to anneal out the radiation damage (heat 1) and then to generate an annealed baseline (heats 2 to 5) to compare to the first heating run. Samples were then heated to 1000°C four times to undergo the α/β phase transition (heats 5 to 8) and measure the enthalpy of transformation. The α/β enthalpy of transformation is −87 ± 4 J/g (*37*). The measured enthalpy was used to validate the instrument sensitivity calibration, which was conducted after the experiments (using a sapphire standard and the NETZSCH *C*_p_ software package). For more details on the calibration procedure, see section S2.2.

DSC data analysis involved fitting a cubic baseline to the areas outside the ROIs and subtracting this to evaluate the specific power at the correct scale (in μW/mg). The effect of the crucible was then corrected for. Heats 2 to 5 (annealed sample) were averaged and subtracted from heat 1 (defected sample) to determine the stored energy released on the first heat. Nine irradiated and nine unirradiated samples were then averaged to increase the signal-to-noise ratio. Error bars show ± the summation in quadrature of the SEs arising from averaging the crucible corrections, heats 2 to 5, and the different samples. The stored energy was evaluated by integrating the signal within each ROI (ROI 1: 380° to 470°C and ROI 2: 500° to 590°C). The uncertainty on the integrals was calculated as the summation in quadrature of SEs arising from averaging the integral from each sample and each correction run. For more details on the analysis procedure, see section S2.1.

### TEM characterization

Samples were annealed to different temperatures in the DSC before preparation for TEM analysis. Four samples were selected: one as-irradiated (*T* = 300°C), one annealed to 480°C (ROI 1 < *T* < ROI 2), one annealed to 600°C (ROI 2 < *T*), and one unirradiated. Each sample then had one TEM lamella prepared using a Tescan Lyra 3 focused ion beam microscope. The sample thickness, and thus defect density, was determined using energy-filtered TEM log ratio method (*38*). The mean free path for inelastic scattering of 200~keV electrons in Ti = 106 nm with an uncertainty of 19% (*39*). For measuring the dislocation loop diameter from the TEM micrographs, ImageJ software was used to determine the Feret diameter.

To correlate the TEM-determined defect densities to the DSC measurements, the energy per dislocation loop was calculated from elasticity theory (*40*, *41*). The energy per length was determined and then multiplied by the dislocation loop size and density to obtain the stored energy density (in J/g). This was compared to the stored energy from DSC integrated over both ROIs (380° and 590°C), with error bars calculated similarly to above. The full details of the calculation are included in section S4.

### MD simulations

#### Displacement cascades

To generate Ti microstructures that were representative of neutron irradiation, MD simulations of consecutive collision cascades were performed using the PARCAS code (*42*). An adaptive time step was used to accurately follow the trajectories of the energetic particles (*43*). The interatomic potential by G. Ackland (*44*) “A92” with close-up repulsion by G. Ackland (*45*) was used, and 10 independent simulations were conducted to increase the statistics of the results. Simulation cells of 492,800 atoms were subject to repeated 5~keV PKAs at 300°C to achieve doses up to 0.6 displacements per atom (8000 PKAs, with a threshold displacement energy of 30 eV). Electronic stopping was active on all atoms with kinetic energy of 5 eV or more. After each cascade was initiated, the simulation cells were held at 300°C for 30 ps to allow for unstable defect configurations to relax. This was done in a two-step manner, first with border cooling not to affect the cascade region, and then, when the cell had equilibrated, a thermostat and barostat on the whole simulation cell to reach a zero overall pressure were applied. The box was randomly shifted after each cascade to obtain a homogeneous irradiation.

#### Defect annealing

Following the PKA cascades, the simulation cells were annealed using the Large-scale Atomic/Molecular Massively Parallel Simulator code (*46*). The simulation cell of 492,800 atoms corresponds to ∼(20 nm)^3^ in volume, and the periodic boundary conditions create an infinite single crystal. The simulations therefore correspond to a system without grain boundaries. Using the NVT ensemble, the defected supercells were relaxed with a 2~fs time step for 5×10^7^ time steps, resulting in a total duration of 100 ns. Supercells were relaxed at 300°, 480°, and 600°C, which respectively correspond to below ROI 1, between ROI 1 and 2, and above ROI 2 in Fig. 1. Cell configurations were output every 0.2 ns and minimized using the conjugate gradient algorithm to relax unstable defect configurations before calculation of the stored energy. The total stored energy was calculated by comparing the potential energy of the defected supercell to that of a pristine crystal. OVITO (*47*) was used to visualize the system with DXA (*48*) analysis used to identify dislocations and WS analysis used to detect point defects (*42*).

## Supplementary Materials

### This PDF file includes:

Supplementary Text Sections S1 to S5 Figs. S1 to S16 Tables S1 and S2 References
