# Supplementary material for: Revealing hidden defects through stored energy measurements of radiation damage
Source: Sci Adv. 2022 Aug 3;8(31):eabn2733. doi: 10.1126/sciadv.abn2733 (PMC9348784; doi:10.1126/sciadv.abn2733)
Supplement: Supplementary file 1 — Supplementary Text Sections S1 to S5 Figs. S1 to S16 Tables S1 and S2 References [file sciadv.abn2733_sm.pdf]

Science Advances
AAAS

Supplementary Materials for

Revealing hidden defects through stored energy measurements of radiation damage

Charles A. Hirst *et al.*

Corresponding author: Charles A. Hirst, cahirst@mit.edu; Michael P. Short, hereiam@mit.edu

Sci. Adv. **8**, eabn2733 (2022)
DOI: 10.1126/sciadv.abn2733

This PDF file includes:

Supplementary Text
Sections S1 to S5
Figs. S1 to S16
Tables S1 and S2
References

Supplementary Text

1. Background

1.1. Limitations of existing characterisation techniques

1.1.1. Transmission electron microscopy (TEM)

TEM is commonly used to image radiation damage by scattering electrons off the locally distorted lattice around defects (22). However, the resolution limit of conventional TEM is typically between 1-2 nm (3). This corresponds to a cluster of ~55 defects for a dislocation loop with diameter of 1 nm in BCC or FCC, or ~350 vacancies for a 1 nm diameter void in BCC (49). The resolution limit thus prevents TEM from characterising point defects and small defect clusters. In addition, not all defects larger than the resolution limit will be visible, due to the $\mathbf{g} \cdot \mathbf{b}$ visibility criterion (50). Simulations and experiments show that the size distribution of irradiation-induced defect clusters follows a power law (2). This means that the majority of clusters in the material are below ~10s of defects in size and thus the majority of clusters may be below the visibility limit for TEM.

With recent developments in aberration-corrected scanning TEM (STEM), it is possible to image radiation damage at high resolution. Liu et al. compare STEM to TEM of Kr-irradiated SiC and show that TEM is unable to detect 2/3 of the defects that are visible in STEM (51). Jiang et al. also observe small defect clusters in electron-irradiated SiC (52). However, for both of these studies, the ability to image the clusters is attributable to the high migration energy and low mobility of carbon interstitials in SiC. This would not be achievable in metals where the interstitial migration energy is over an order of magnitude lower. The thickness requirements for S/TEM samples result in free surfaces which act as sinks for mobile defects. Therefore, in metals, up to the entire sample may be considered to be a denuded zone and point defects may no longer be present. The mobility of defects scales inversely with cluster size (53), thus the defects that most likely to occur are both unable to be imaged in TEM and also least likely to be present in the sample.

Many studies have shown that the limitations of TEM prevent it from characterising the full population of defects in a sample. Meslin et al. report a defect number density that is an order of magnitude lower for TEM than for small angle neutron scattering (SANS) or positron annihilation spectroscopy (PAS) of neutron-irradiated Fe (4). Reza et al. observe a similar scale discrepancy between TEM and transient grating spectroscopy (TGS) of self-ion irradiated W (5). Song et al. also report an order of magnitude between defect number densities determined by TEM and lattice strain measurements of Fe^{3+} irradiated FeCr (54).

Vastly underestimating the defect density prevents TEM from accurately determining the corresponding change in properties. Weiß et al. show a factor of 2 between measured and calculated (from TEM) change in hardness for neutron irradiated EUROFER (55). Reza et al. report the same discrepancy between TGS-measured and TEM-determined thermal diffusivity for self-ion irradiated W (5). Notably, when Reza et al. account for small defects through the addition of results from molecular dynamics (MD) simulations, the combination of TEM and MD matches TGS measurements. This result confirms the theory that point defects play a significant role in the thermal diffusivity of a material and further reinforces the need to accurately characterise small defects in order to evaluate irradiation-induced changes in properties. It also highlights the inability of TEM to validate radiation damage simulations as the true number of Frenkel pairs cannot be determined by electron microscopy.

1.1.2. Resistivity measurements

Interpretation of resistivity measurements in metals requires solving Matthiessen's rule, an equation which details the electron scattering contribution from various defects as a function of their size and density (56). A consequence of this is the sublinear relationship between resistivity and cluster size (57), which reduces the sensitivity of these measurements to larger defects at lower concentrations. As such, resistivity measurements are well suited for characterising isolated point defects at low temperatures (such as those resulting from cryogenic electron irradiation) but less well suited to characterisation of larger defects (such as dislocation loops) following neutron irradiation at reactor-relevant temperatures.

1.2. Stages of radiation damage recovery

The evolution of defects in metals is strongly linked to temperature. The difference in migration energy between interstitials and vacancies leads to characteristic stages of radiation damage recovery as a function of temperature (20). For

the case of a metal irradiated close to absolute zero, initially all defects are 'frozen' in the lattice. As the temperature increases, the following defect reactions occur:

- Stage I - following recombination of close Frenkel pairs, interstitials become mobile. They migrate and either annihilate with vacancies or cluster with other interstitials.
- Stage II - increasing interstitial mobility leads to the growth of interstitial clusters into small dislocation loops.
- Stage III - vacancies become mobile and can annihilate at interstitial clusters. Vacancy clustering also occurs, resulting in a microstructure containing small vacancy clusters and larger interstitial loops.
- Stage IV - both interstitial loops and vacancy clusters grow with increasing temperature.
- Stage V - vacancy clusters become thermally unstable, emitting single vacancies. These migrate to interstitial loops and annihilate.

These stages of recovery occur at approximately the same fixed fraction of the melting point for metals of a given crystal structure. For the FCC metals Al, Cu, Au, Ni, stage III occurs at 220, 250, 290 and 340 K respectively, this corresponds to ∼0.21 T_m. Stage V recovery occurs at 420, 550, 650 and 760 K respectively, which corresponds to ∼0.45 T_m (58). Prior resistivity studies have explored this recovery mechanism in detail, revealing further substages (59). Stage I contains contributions from recombination of close, correlated and uncorrelated Frenkel pairs. Stage II exhibits a substage which may be related to the migration of small interstitial clusters. These studies show that great insight into a material's structure can be obtained through detailed annealing experiments.

The above recovery model was developed from cryogenic irradiations, often using electrons. The picture becomes more complex for neutron irradiation due to the intracascade formation of defect clusters at temperatures below stage I. In addition, the presence of solutes can result in trapping of defects, resulting in enhanced stages II and IV (20). The effect of pre-existing defects is also not considered, with the assumption that dissociated point vacancies migrate to interstitial clusters and annihilate rather than recombine at dislocation lines or grain boundaries.

1.3. Wigner energy in metals

The concept of Wigner energy has more commonly been applied to ceramics than metals. Radiation damage in ceramics can lead to the formation of amorphous regions (60). Due to the large number of displaced atoms within these regions, ceramics can store energy values of the order 10^3 J/g (14). The large values of stored energy result in easily detectable annealing peaks in differential scanning calorimetry (DSC) experiments. Radiation damage in metals, on the other hand, takes the form of Frenkel pairs and their clusters (60). The smaller number of defected atoms in metals leads to stored energy values typically less than 1 J/g (14). As a result, investigating defect annealing using DSC is more challenging for metals than in ceramics.

While most defect annealing studies have focused on cryogenic irradiations, experiments following room temperature irradiation have been conducted. These include Cu (33, 61), Mo (61, 62), W (62), and Ni and Fe-based alloys (63). However, very few studies have been performed after irradiation at higher, reactor-relevant, temperatures (64, 65).

One of the advantages of stored energy over resistivity is its ability to be simulated for systems up to $\sim10^6$ atoms. Insight into the precise mechanism of energy release can be gained through simulated defect annealing. MD (19) and kinetic Monte Carlo (kMC) (66) simulations are often used to evaluate defect evolution as a function of temperature. Changes in the population of defects and the resulting potential energy difference can be readily determined through these methods. This knowledge can be used to aid interpretation of the defect reactions measured in DSC experiments.

1.4. Prior TEM characterisation of irradiated Ti

TEM has previously been used to study radiation damage in Ti following mixed-spectrum neutron (67, 68), fast neutron (21, 69), and dual-ion irradiation (70). Irradiation between 300-400°C forms a high density of <**a**>-type dislocation loops. The majority of these dislocation loops are vacancy-type and have a diameter smaller than that of the interstitial-type loops (68). This microstructure is consistent with that corresponding to Stage III recovery in metals. In some cases, it is reported that the interstitial loops have grown large enough to intersect and form network dislocations (21). After irradiation at 430°C, the ratio of vacancy to interstitial loops decreases and at higher irradiation

temperatures the predominant form of damage is a low density of <**a**>-type network dislocations (21). Following irradiation at 630°C, there are no dislocations observed with damage in the form of small defect clusters (70). However, in this study this microstructure may have been influenced by the implantation of helium that would stabilise the formation of small defect clusters. The absence of dislocations at these temperatures is consistent with cold work recovery of Ti, which indicates that dislocations become mobile above 550°C (71, 25).

2. DSC methods

2.1. Data analysis process

In order to evaluate the stored energy released during annealing, the raw data in $(\frac{\mu V}{mg})$ must be converted to $(\frac{mW}{mg})$ and integrated to yield an energy density in $(\frac{J}{g})$. The full analysis process is depicted in figure S1. The steps are as follows:

0. The raw DSC data is measured in $(\frac{\mu V}{mg})$. This is then divided by the sensitivity in $(\frac{\mu V}{mW})$ to yield the commonly used DSC units of $(\frac{mW}{mg})$. For additional details on the sensitivity calibration see section S2.2.

1. Visually inspecting the DSC data in the temperature range of interest (300°C $< T <$ 600°C) shows there are no obvious enthalpic reactions. Note that the y-axis scale is in $(\frac{mW}{mg})$. Estimating the expected peak height, assuming a stored energy density of 0.1 J/g, an energy release peak width of 50°C, and a heating rate of 50°C/min gives an expected peak height of the order $(\frac{\mu W}{mg})$. This is impossible to resolve at the current y-axis scale and therefore the data must have a baseline fitted and subtracted.

2. For each individual heating run, a cubic polynomial baseline is fitted to the data between 300–350, 450–500, and 575–600°C and then subtracted from all the data between 300–600°C. This allows comparison between subsequent runs, note that now the y-range is now in $(\frac{\mu W}{mg})$. For additional details regarding the baseline determination see section S2.3.

3. At this stage the data still represents the signal from the crucibles, the samples, and any enthalpic effects within the sample. In order to correct for the effect of the crucibles, the signal measured from empty crucibles (subject to the same heating profile) is subtracted from the data. The error bars are ± standard error of the signal from the crucibles, which is the average of 20 heating runs. For more details about the correction procedure refer to section S2.4.

4. To generate a physical baseline from the annealed sample, heating runs 2–5 are averaged. The error bars are now the summation in quadrature of a) the standard error from the averaging of the crucible corrections, and b) the standard error from the averaging of heating runs 2–5.

5. The annealed baseline is then subtracted from the first heating run to determine the DSC signal arising from irreversible changes to the sample during heat 1. Exothermic peaks have become evident.

6. In order to increase the signal-to-noise ratio and to confirm the reliability of the results, 9 identical samples were measured and their signals averaged. The error bars now show the summation in quadrature of the previous contributions plus the standard error arising from averaging the different samples.

7. The same procedure was conducted for 9 unirradiated samples sectioned from an identical Ti nut. The unirradiated samples do not exhibit significant exothermic peaks.

8. The specific power was integrated as a function of time over the temperature ranges 380–470 and 500–590°C. This yields values of the stored energy released during annealing in $(\frac{J}{g})$. The integral limits were chosen as the temperatures where the signals diverged for the irradiated and unirradiated samples.

The uncertainty on the integral values of stored energy in figure 1 is calculated by integrating the signal from the individual samples and calculating the standard error from the values of stored energy, integrating the signal from the correction runs and calculating the standard error of these values, and summing both these errors in quadrature.

2.2. *Sensitivity calibration*

To convert DSC data from $(\frac{\mu V}{mg})$ to $(\frac{mW}{mg})$ the sensitivity of the DSC must be determined. This is done by heating a sapphire sample (1.0 mm thickness, 4 mm diameter) and comparing the measured signal to the reference values for heat capacity. Figure S2a shows the raw DSC data in $(\frac{\mu V}{mg})$. At discrete points this is divided by the specific heat capacity to yield the 'Exp.' sensitivity data in $(\frac{\mu V}{mW})$ shown in figure S2b. In order to determine the sensitivity function across all temperatures, the data is fit to the following equations,

$$\text{Sensitivity} = f(T) = \sum_{i=1}^{4} P_{i+1} z^{i-1} e^{-z^2} \tag{1}$$

$$z = (T - P_0)/P_1 \tag{2}$$

where T is the temperature and P_{0-5} are coefficients, shown in table S1. This function is plotted as the 'Calc.' line in figure S2b. Note these coefficients are the mean of values from three sensitivity calibrations.

P_0	P_1	P_2	P_3	P_4	P_5
223.1	683.4	4.315	0.174	-5.895	4.706

Table S1: Sensitivity calibration coefficients.

2.3. *Baseline determination*

The inherent range of the DSC data in $(\frac{mW}{mg})$ is much greater than the expected peak height. To resolve these small peaks a baseline was fitted to the DSC data and subtracted. A cubic polynomial was chosen as the lowest order function to capture the macroscopic characteristics of the data and has also previously been used to model the heat capacity of Ti (*72*, *73*).

Initially the baseline was fitted to all the data between 300–600°C, see figure S3a. This resulted in an offset of the irradiated data, seen in figure S3b. Considering the fitting of a polynomial function to a curve containing enthalpic peaks, the presence of peaks would result in a baseline that is displaced from the true datum. In the case of exothermic peaks, the baseline would be displaced in the positive direction which, when subtracted from the function, would lead to resultant data that is artificially displaced downwards. In order to determine the temperatures at which enthalpic reactions may occur, prior literature was consulted. PAS studies annealing neutron-irradiated Ti show a decrease in the vacancy-type defect density between 350–450°C (*24*). Additionally, previous DSC studies of cold-work recovery in Ti report dislocation recovery occurring at 530°C (*26*). Therefore to obtain a true baseline for the DSC curves, a cubic polynomial was fit to the data between 300–350, 450–500, 575–600°C, see figure S3c. This results in processed data in which the irradiated and unirradiated curves match each other outside the regions of interest, and also approximately equal zero specific power, see figure S3d.

2.4. *Crucible correction*

To correct for the effect of the crucibles and instrument, empty crucibles were heated through the same temperature profile as the samples (so-called 'correction' runs). The signal was subject to the same baseline process, and then was subtracted from the sample data. In DSC artefacts are common; these can arise from a slight shifting of the crucibles which changes the thermal contact between the crucibles and the sensor. Therefore, in order to ensure that such artefacts were not introduced into the sample data, 20 corrections runs were conducted and then averaged before subtraction. This is shown in figure S4.

2.5. α/β calibration

Sensitivity calibration of the DSC is carried out periodically. Therefore, to ensure confidence in the DSC results, experiments were designed to utilise the built-in enthalpy calibration. Ti exhibits a phase transformation from α-BCC to β-HCP at 893°C with a specific enthalpy of -87.1 ± 4.4 J/g (37). After annealing the radiation damage the Ti samples were heated to 1000°C 4 times, shown in figure S5a.

Heat 5 was not used for determination of the α/β enthalpy as there may be retained β-phase from processing. Heats 6 to 8 were each corrected for the effect of the crucible, and a linear baseline was fit to the data between 600–800°C and 970–990°C and subtracted. The data was then integrated between 820–980°C to determine the enthalpy of phase transformation. This process can be seen in figure S5b. The enthalpies from heats 6 to 8 were then averaged to improve the accuracy of the measurement. The uncertainty stated is the standard deviation of the averaged enthalpy and the small magnitude of this value confirms the reliability of the measurement. This can be seen in figure S5c.

The average specific enthalpy of the α/β transformation is plotted for all samples in figure S5d. The nomenclature is as follows: samples A-, B-, and C- are irradiated, and samples U- are unirradiated. The samples are plotted in chronological order and it can be seen that there is no consistent drift of the values over time. This confirms that the sensitivity calibration is valid for all samples.

2.6. Uncertainty evaluation

For the three instances where multiple runs are averaged (corrections, heats 2–5, 9 samples) the uncertainty can be evaluated as a function of iteration to determine if the number of repeats is sufficient. At each temperature, the standard error of the DSC signal is calculated as a function of the number of runs/samples. This is then averaged across the whole temperature range to give one value of standard error as a function of the population size. This is shown in figure S6.

For the correction runs, the mean standard error starts to a plateau after 15 corrections runs. This indicates that 20 corrections is sufficient to minimise the uncertainty from this contribution. Next, analysing the mean standard error as a function of heats, the uncertainty increases from the second to third sample heat (first to second annealed heat), as expected due to the inherent variation between runs, but then is approximately constant with increasing number of sample heats. This shows that the baseline does not strongly depend on the number of heats and would not be improved with further iteration. Finally, looking at the uncertainty with respect to the number of samples averaged, initially there is an increase for 2–5 samples, followed by a decrease from 5–9 samples. The notable increase between samples 4 and 5 may be due to the 5th sample being dissimilar to previous ones, however this effect is mitigated when averaging over 9 samples. The mean standard error is still decreasing at 9 samples, but the uncertainty is approaching the magnitude of the lowest values observed. Constraints on the availability of neutron-irradiated samples present an upper limit on the number of samples that can be measured, which ultimately determined the minimum uncertainty that can be achieved.

The sensitivity calibration used was the average of sensitivity functions derived from 3 consecutive heats of the sapphire standard. The mean values and variation are shown in figure S7a. The difference between the experimental data and calculated function gives a measure of the uncertainty, shown in figure S7b. While the largest relative difference is ~12%, which is likely due to the decreasing value of sensitivity with temperature, in the temperature range of the experiments (40–1000°C) the greatest uncertainty is ~8%.

One other evaluation of the uncertainty is the difference between the measured enthalpy of transformation $\Delta H_{\alpha/\beta}^{avg.}$ and the reference value. These are shown in figure S7c where it can be seen that the maximum uncertainty is 19% (for sample 'UR2-3').

3. TEM methods & analyses

3.1. Additional images & loop size distribution

Additional scanning transmission electron microscopy (STEM) and TEM images can be seen in figures S8, S9, S10, and S11 for the irradiated, annealed, and unirradiated samples. The as-irradiated sample and the irradiated & annealed to 480°C sample have similar microstructures with <**a**>-type dislocation loops and linear dislocations, as shown in figures S8 and S9. Furthermore, the size distribution of the dislocation loops between these samples are similar, as

shown in figure S12. In contrast, the irradiated & annealed to 600°C sample exhibits a typical recovered microstructure with dislocation substructures, sporadic distribution of line dislocations and no dislocation loops. This is similar to the microstructure of unirradiated sample (see figures S10 and S11). Such microstructure with dislocation substructures and linear dislocations has previously been observed in dynamically recovered hexagonal closed packed (HCP) alloys (74).

The dislocation loops have been identified as <**a**>-type by indexing the TEM images. Figure S8 shows that the dislocation loops are elliptical with major axis ∥ to **c** and minor axis ∥ to the **a** axis. This is in agreement with multiple studies from prior literature (67, 68). Regarding the presence of <**c**>-type dislocation loops, previous literature reporting on neutron irradiation of Ti under similar conditions (our work: T_{irr}=300°C and fluence ($>$ 0.1 MeV) = 6.3×10^{20} cm^{-2}, Brimhall et al. (67): T_{irr}=450°C and fluence ($>$ 0.1 MeV) = 3×10^{21} cm^{-2}, Jostsons et al. (68): T_{irr}=400°C and fluence ($>$ 1 MeV) = 3.9×10^{19} cm^{-2}, and Griffiths et al. (21): T_{irr}=316°C and fluence ($>$ 0.1 MeV) = 1.7×10^{21} cm^{-2}) has found that radiation damage consists only of <**a**>-type dislocation loops. Thus <**c**>-type dislocation loops are not expected to be produced in our samples. Furthermore, if <**c**>-type dislocation loops (with $\mathbf{b} = \frac{1}{6}\langle 20\bar{2}3\rangle$) were present, the imaging conditions used in our study would result in them being visible as the product of **g**·**b** is non-zero.

3.2. Defect density quantification

The number density and size distribution of dislocation loops (from TEM images of the as-irradiated sample) were used to calculate the energy stored in dislocation loops. ImageJ image analysis software was used to count the number (N) of dislocation loops and obtain their size using tracing method as shown in figure S13. The Feret diameter of the dislocation loops was used as a measure of the dislocation loop size. The thickness of the TEM lamellae, at the locations of the images, was determined using energy-filtered TEM (EFTEM) maps shown in figure S14. The EFTEM maps provide the ratio between the TEM specimen thickness (t) and the inelastic mean free path (λ) of electrons at 200 keV. The λ value for Ti at 200 keV and the uncertainty in its estimation are 106 nm and 19%, respectively, obtained from literature (39). The thickness of the TEM lamellae was used to determine the volume imaged (V_T), and thus calculate the dislocation loop number density (D_{loop}) using equation 3. The error in the dislocation loop number density calculation was estimated from the uncertainty in the TEM thickness measurement.

$$D_{loop} = N/V_T \tag{3}$$

Comparing our calculated number density of loops to previous literature shows that our values match closely. Brimhall et al. (67), Jostsons et al. (68), and Griffiths et al. (21) report loop number densities of 2.5×10^{21} m^{-3}, (7±1)×10^{20} m^{-3}, and 7×10^{20} m^{-3}, respectively, compared to our value of (4.0±0.7)×10^{21} m^{-3}. The match between these values establishes confidence that our measurements are accurate.

To further confirm the accuracy of our TEM experiments we conducted analysis of an image taken from the same grain under different imaging conditions. In figure S15, the [$\bar{1}$10] zone axis and **g** = 110 were used. It is noted that, should there be any, <**c**>-loops would not show contrast under these imaging conditions. Despite this, the number density of dislocation loops calculated from the image is (within uncertainty) the same as previously determined, namely (4.4±0.8)×10^{21} m^{-3}. Additionally, analysis of an image from a different grain taken under these imaging conditions yields a very similar number density of (4.1±0.8)×10^{21} m^{-3}. That the dislocation loop density is consistent under different imaging conditions and at different locations provides confidence in the accuracy of our results.

The areal number density of linear dislocations in the as-irradiated sample and the sample irradiated and annealed to 480°C was determined as (2.4±0.5)×10^{13} m^{-2} and (3.2±0.6)×10^{13} m^{-2}, respectively. This contributes a stored energy density of less than 9% when compared to the dislocation loops. Annotated images are shown in figure S16. Additionally, so-called 'black spot defects' were determined to have a number density of $(5.0 \pm 0.9) \times 10^{20}$ m^{-3} and average size of 3 nm. These contribute a stored energy density of less than 2% when compared to the dislocation loops.

4. Dislocation loop energy calculations

To make a comparison between DSC and TEM measurements the energy per defect must be determined. Inspired by recent work by Liu et al. (*41*), elasticity theory was used to calculate the energy for an elliptical <**a**>-type dislocation loop on the prismatic plane in Ti. In order to accurately calculate the dislocation loop energy, the effects of temperature and anisotropy were accounted for. Temperature-dependent elastic constants for Ti were obtained from Fisher et al. (*75*). The temperature of most relevance to our experiments is 550°C which corresponds to the peak temperature of the second annealing peak observed in the DSC. The following equations and the parameters detailed in table S2 were used to calculate the energy per loop as

$$E_{loop} = 2\pi R \cdot K \cdot \frac{b^2}{4\pi}\left(\ln\left[\frac{4R}{(b/2\alpha)\cdot\exp(\gamma)}\right] - 1\right) \tag{4}$$

Variable	Description	Value	Ref.
R	Radius of dislocation loop	9.5 nm	this work
b	Burgers vector of prismatic loop	2.95 Å	(*76*)
α	Dislocation core parameter	1.25	(*40*)
exp(γ)	exp(γ) = exp(1-2ν)/4(1-ν)	1.17	(*40*)

Table S2: Parameters used for dislocation loop energy calculation.

4.1. Anisotropy dependence

Parameter K is the energy factor which contains the temperature-dependent elastic constants and orientation dependence of the dislocation line. From Savin et al. (*77*), the energy factor for an edge dislocation on the prismatic plane, aligned along the **c** axis with **b**=$\frac{1}{3}\langle 1\bar{2}10\rangle$, is given by

$$K_{\mathbf{c}} = \frac{C_{11}^2 - C_{12}^2}{2C_{11}} \tag{5}$$

The energy factor for an edge dislocation on the basal plane, with **b**=$\frac{1}{3}\langle 1\bar{2}10\rangle$, is given by

$$K_{\mathbf{a}} = (\lambda^2 C_{33} + C_{13}) \times \left[\frac{C_{44}(\lambda^2 C_{33} - C_{13})}{C_{33}(\lambda^2 C_{33} + C_{13} + 2C_{44})}\right]^{\frac{1}{2}} \tag{6}$$

where

$$\lambda^2 = \left(\frac{C_{11}}{C_{33}}\right)^{\frac{1}{2}} \tag{7}$$

Evaluating these expressions at 550°C gives energy factors of $K_{\mathbf{c}}$= 31.0 GPa and $K_{\mathbf{a}}$= 53.2 GPa and a ratio of $K_{\mathbf{c}}/K_{\mathbf{a}}$ = 0.58. These calculations are supported by Brimhall et al. (*67*) who determine the same ratio to be $K_{\mathbf{I}}/K_{\mathbf{III}}$ = 0.57, demonstrating an excellent match between our calculations and prior literature. From our TEM experiments, the major (|| to **c**) and minor (|| to **a**) axes of the 244 observed elliptical dislocation loops in the as-irradiated sample are averaged to give a mean ratio of 1.70 ± 0.03 (± standard error). This results in a weighted average energy factor of

$$K_{eff} = \frac{1.7}{1.7+1} \times K_{\mathbf{c}} + \frac{1}{1.7+1} \times K_{\mathbf{a}} = 39.2\ GPa \tag{8}$$

Combining this value with equation 4 resulted in an energy per length of 7.8 $\frac{\text{eV}}{\text{nm}}$ and a total energy per dislocation loop of 467 eV.

5. MD methods & analyses

5.1. PKA and annealing input files

Sample input files for the PKA simulations and instructions how to run them are provided in the data repository: https://github.com/shortlab/2021-Ti-Wigner. Also included are input files for the annealing simulations and all the atomic configurations before and after annealing at 300, 480, and 600°C. The 'before' configurations are 10 unique supercells, each containing 492,800 atoms, which have seen 8000 PKAs corresponding to a dose of 0.6 dpa.

5.2. Videos of annealing simulations

Videos showing the annealing of radiation damage at 300, 480, and 600°C are included in the data repository: https://github.com/shortlab/2021-Ti-Wigner

FIGURES - SUPPLEMENTARY

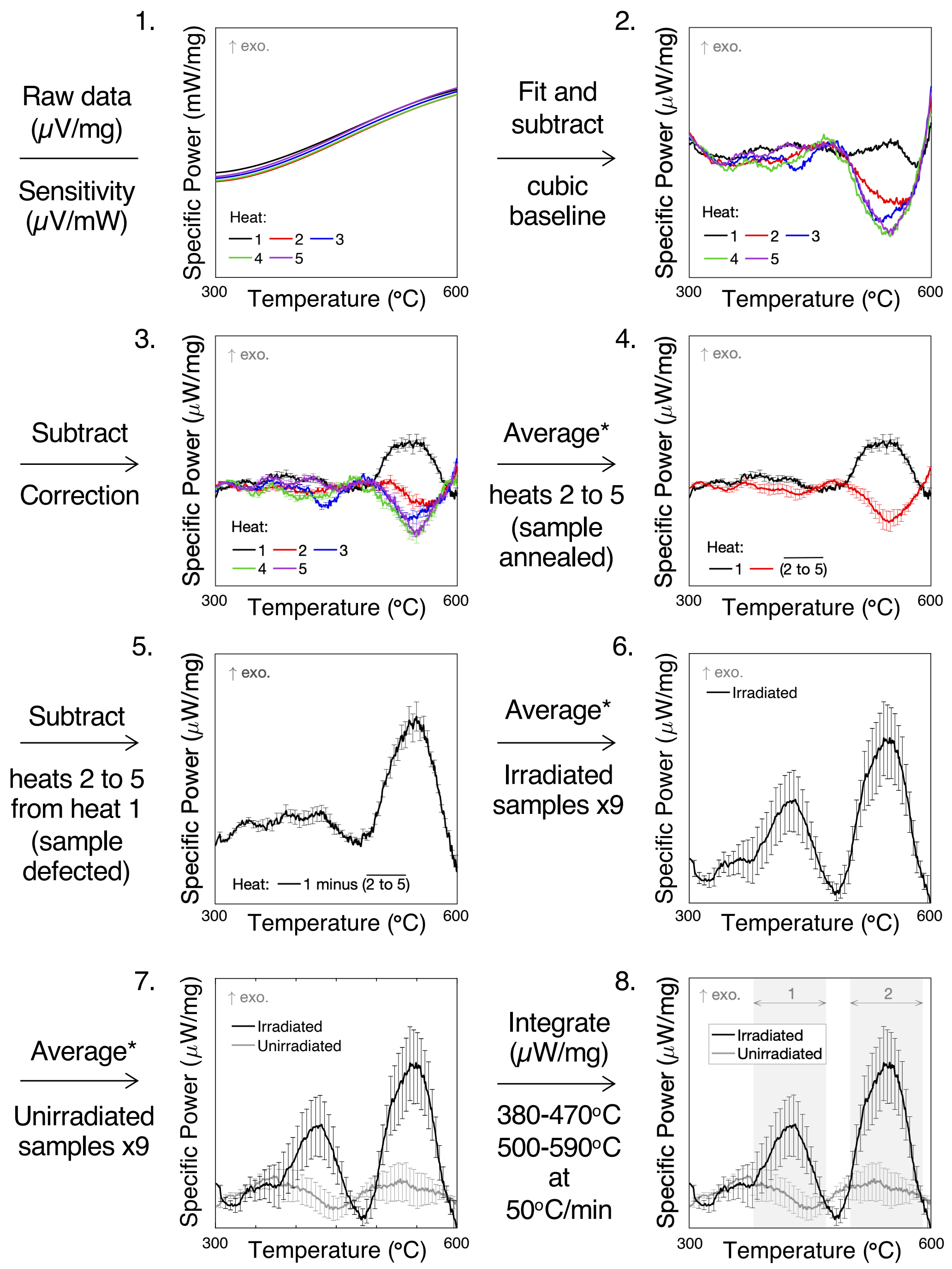

Figure S1: DSC data is analysed to convert from raw data in ($\frac{\mu V}{mg}$) to an energy density in ($\frac{J}{g}$). *After averaging, the standard error is propagated through all subsequent steps, contributions are summed in quadrature.

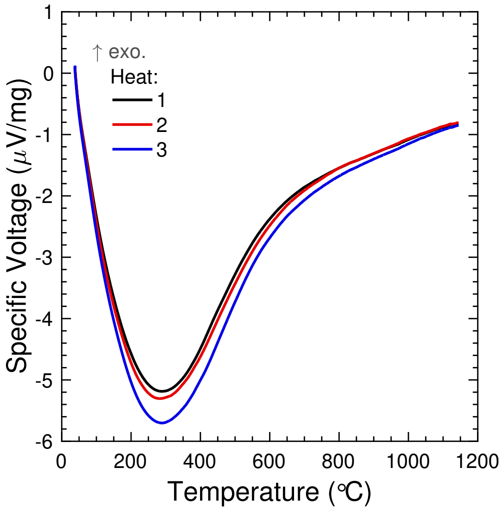

(a) C_p standard DSC measurement.

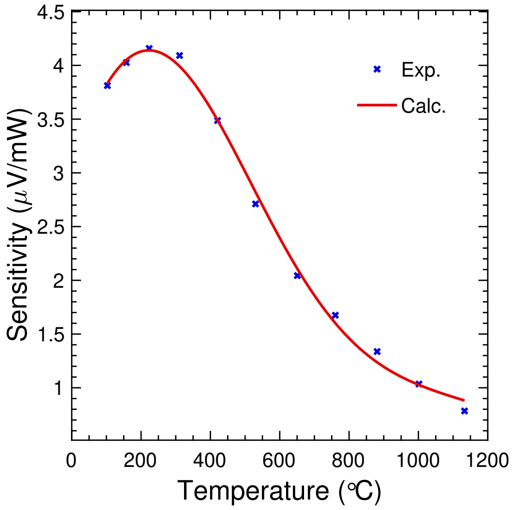

(b) DSC sensitivity calibration function.

Figure S2: Sensitivity calibration allows the conversion of measurements in $(\frac{\mu V}{mg})$ to $(\frac{mW}{mg})$.

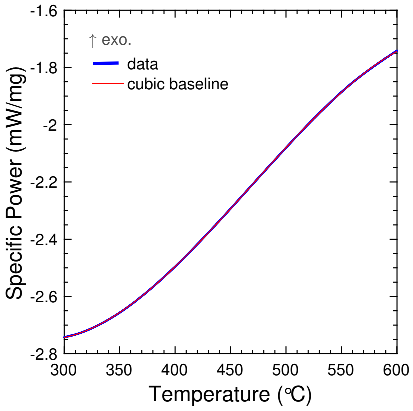

(a) A baseline is fitted to all the data between 300–600°C.

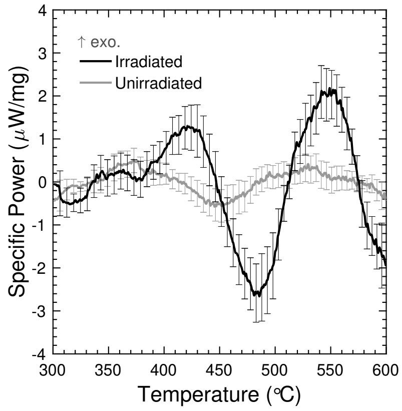

(b) Fitting a baseline to data containing possible exo/endothermic peaks results in curves which are shifted in the y-direction.

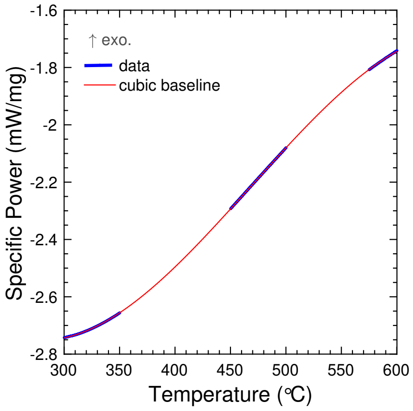

(c) A baseline is fitted to the curve outside the regions of interest (ROIs), data between 300–350, 450–500, 575–600°C.

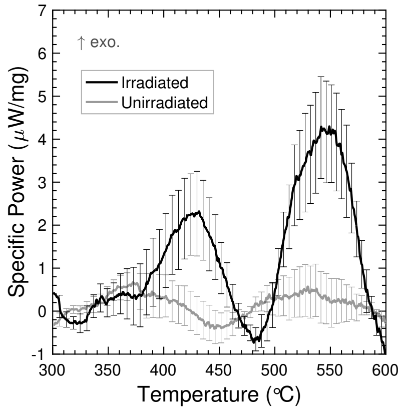

(d) Excluding the ROIs from the baseline fit results in a curve where I≈U≈0 outside of ROIs.

Figure S3: The baseline is fit to data outside the ROIs to avoid artificially shifting the curves in the y-direction.

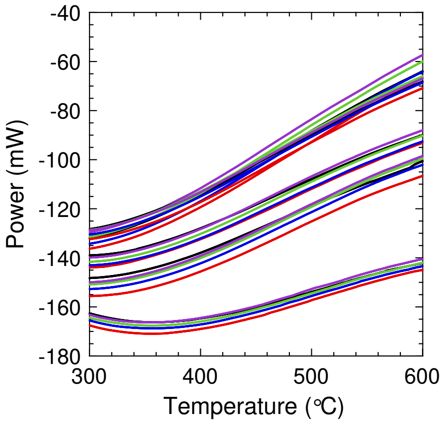

(a) Empty crucibles are subject to the same heating profile as the samples.

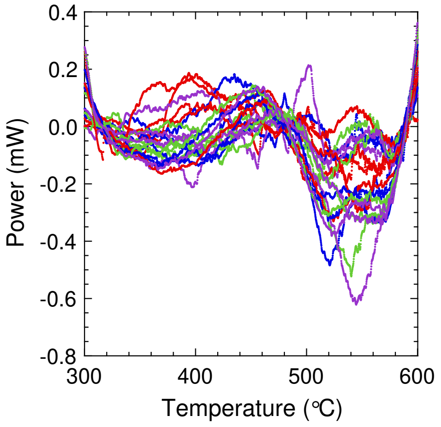

(b) The data is then analysed in the same way as the sample runs.

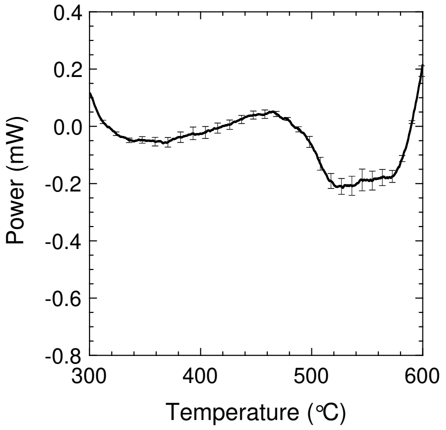

(c) The signal arising from the crucibles can then be subtracted from the samples.

Figure S4: Correction runs allow the effect of the crucibles and instrument to be subtracted from the data.

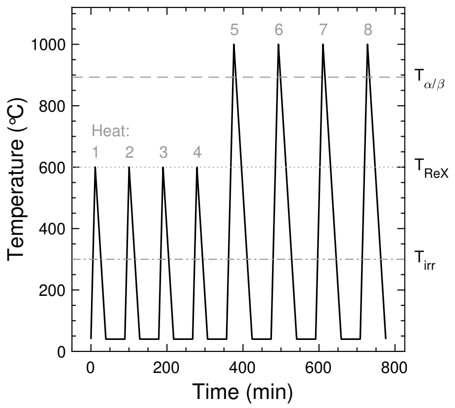

(a) DSC heating profile. Heats 5–8 undergo α/β transition. T_{ReX} = recrystallisation temperature.

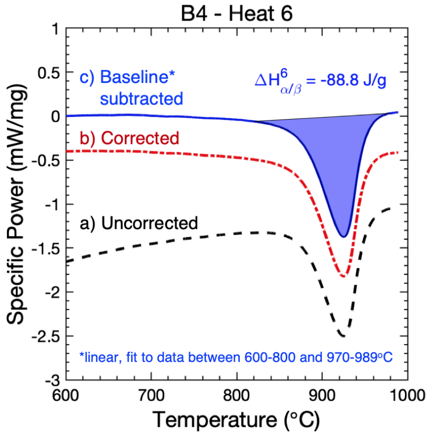

(b) Data analysis process for $\Delta H_{\alpha/\beta}$.

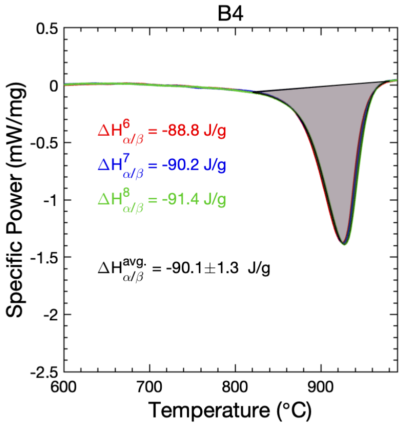

(c) Heats 6–8 are averaged to determine $\Delta H^{avg.}_{\alpha/\beta}$. Instrument reliability heat-heat is confirmed.

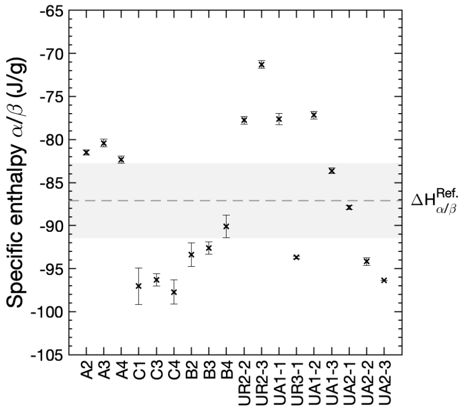

(d) $\Delta H^{avg.}_{\alpha/\beta}$ for all samples in chronological order. Shows that instrument calibration does not drift.

Figure S5: Enthalpy of α/β phase transformation is used to confirm DSC sensitivity calibration.

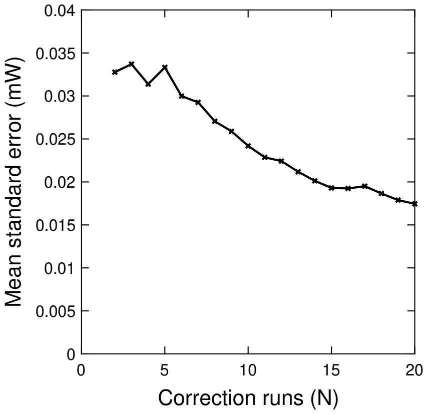

(a) 20 correction runs with empty crucibles were averaged.

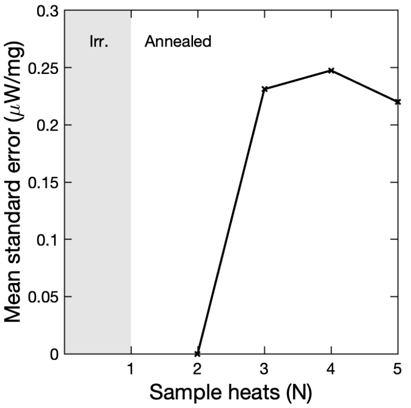

(b) Heats 2–5 are averaged to form the annealed sample baseline.

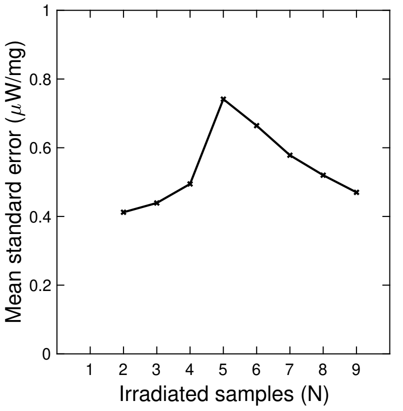

(c) 9 irradiated samples were averaged to increase the signal to noise ratio.

Figure S6: DSC uncertainty is reduced by averaging multiple repeats. For all plots the standard error of the DSC signal is calculated at each temperature, as a function of the number of runs, then averaged over the full temperature range.

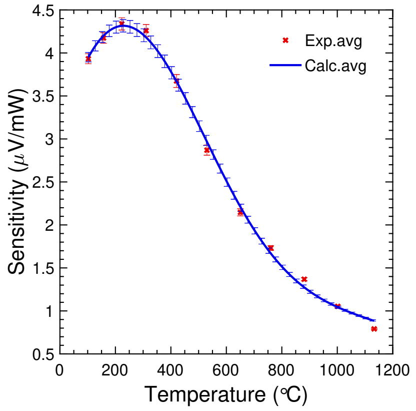

(a) The sensitivity calibration is formed from the average of 3 heats. Error bars show $\pm$ the standard error.

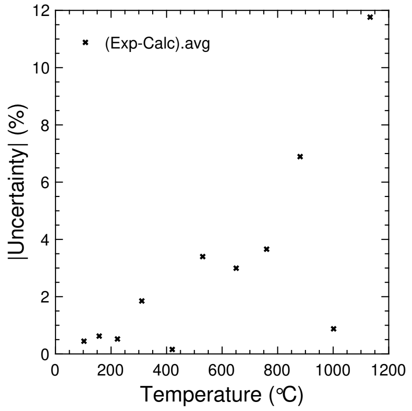

(b) The maximum uncertainty of the sensitivity calibration in the temperature range of experiments is $<8\%$.

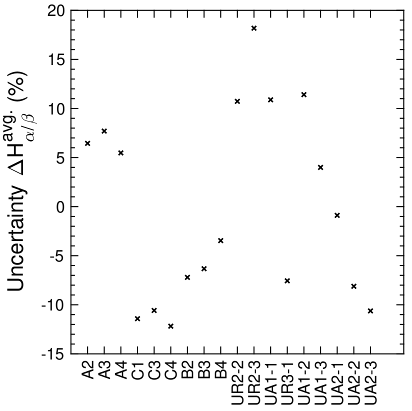

(c) The α/β calibration allows the sensitivity uncertainty for *each* sample to be determined. The maximum uncertainty is 19%.

Figure S7: DSC uncertainty in sensitivity is less than 19%.

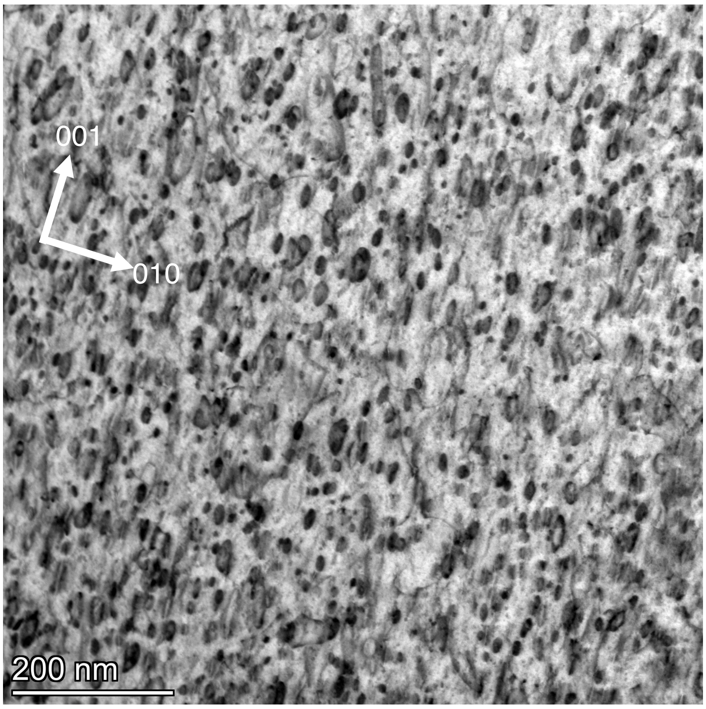

(**a**) STEM

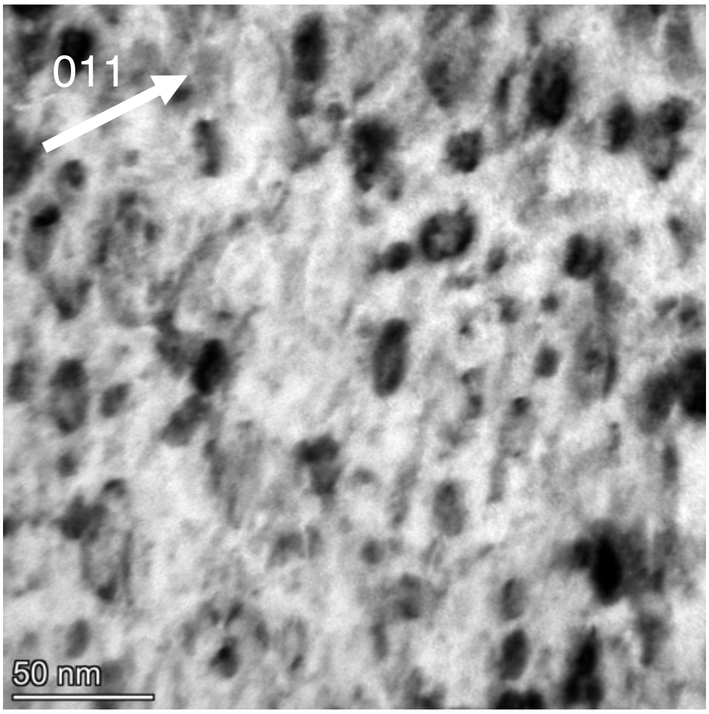

(**b**) TEM

Figure S8: Full-scale, indexed, and additional images of the as-irradiated sample. The diffraction conditions for both images were the two-beam condition with g = 011 and zone axis = [100]. The indexed image shows that the elliptical dislocation loops have a major axis || to **c** and minor axis || to the **a** direction, in agreement with prior literature.

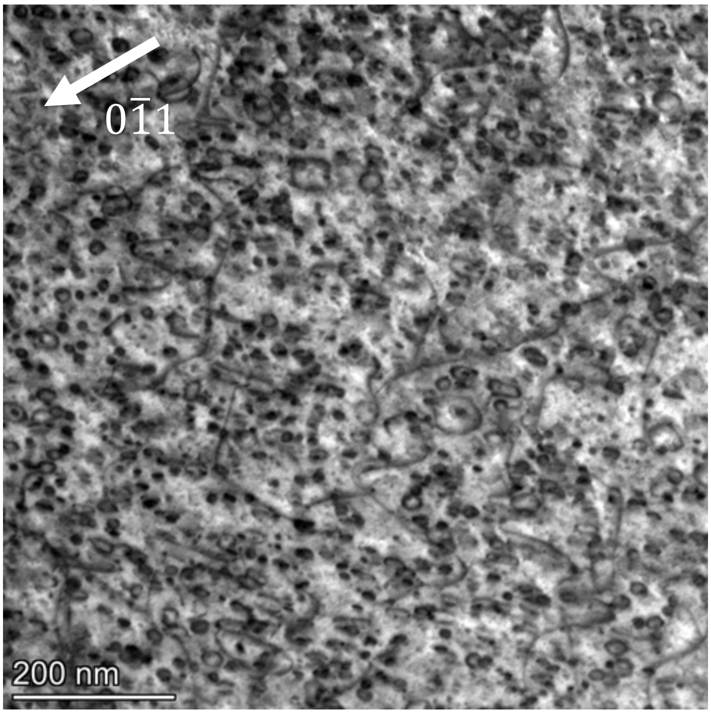

(a) STEM

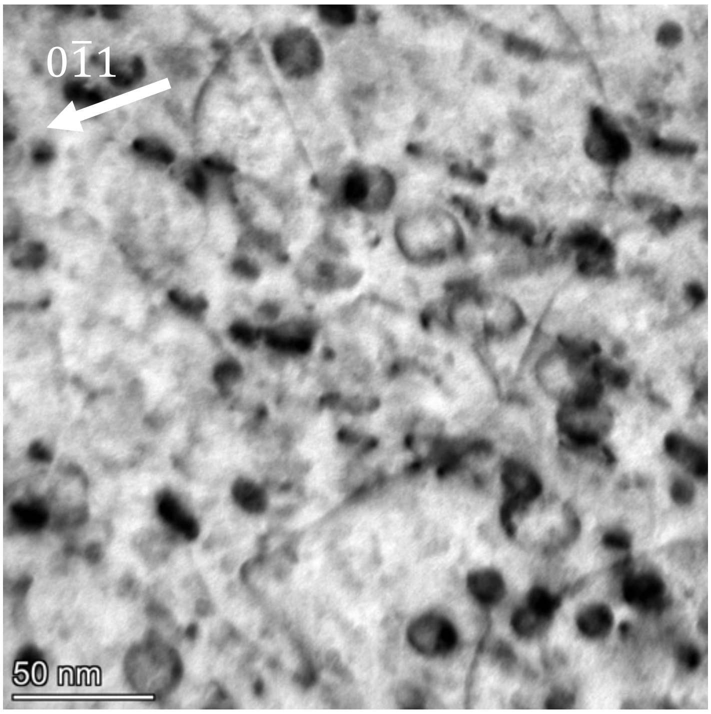

(b) TEM

Figure S9: Full-scale and additional images of the sample irradiated and annealed to 480° C. The diffraction conditions for both images were the two-beam condition with g = $0\bar{1}1$ and zone axis = [311].

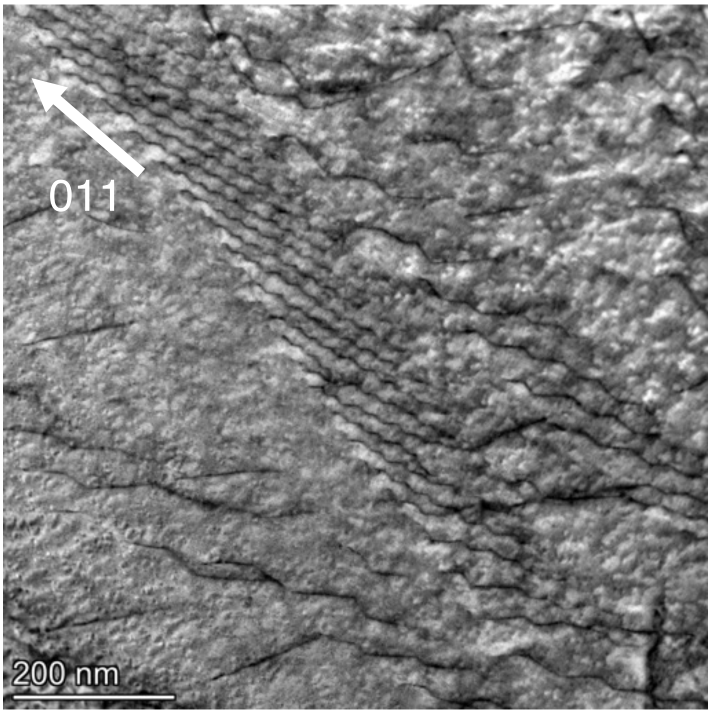

(a) STEM

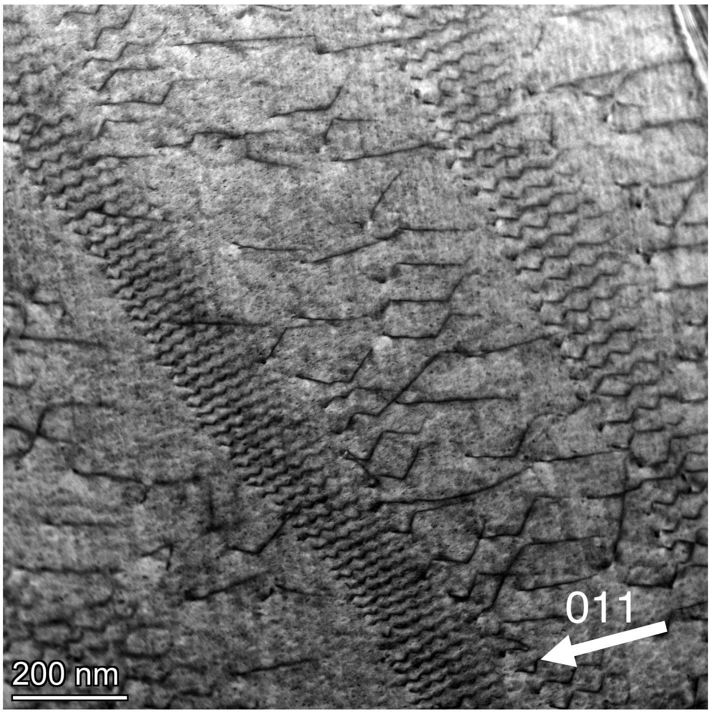

(b) STEM

Figure S10: Full-scale and additional STEM images of the sample irradiated and annealed to 600°C. The diffraction conditions for both images were the two-beam condition with g = $01\bar{1}$ and zone axis = [111].

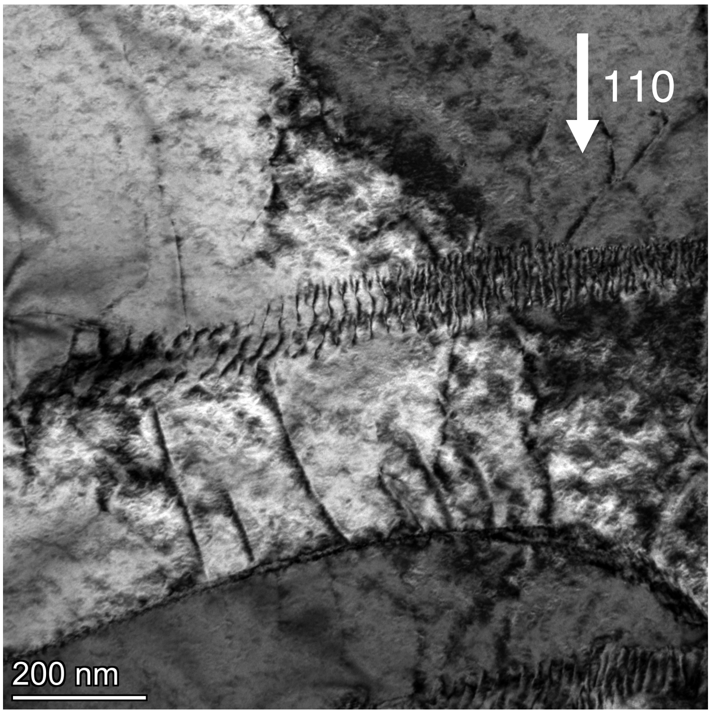

(a) TEM

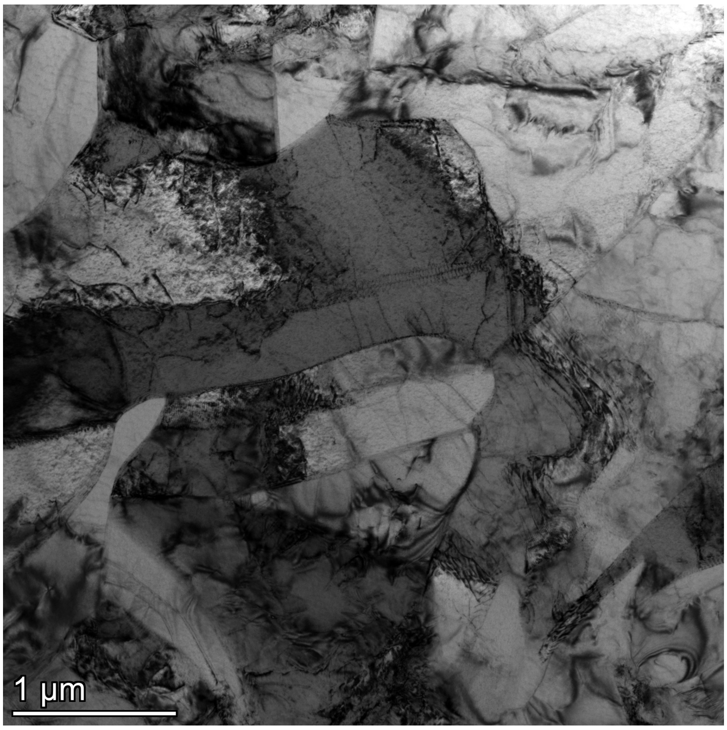

(b) TEM

Figure S11: Additional TEM images of the unirradiated sample. Image a) was taken in the two-beam condition with g = $1\bar{2}0$ and zone axis = [210]. Image b) contains several grains and thus does not have defined diffraction conditions.

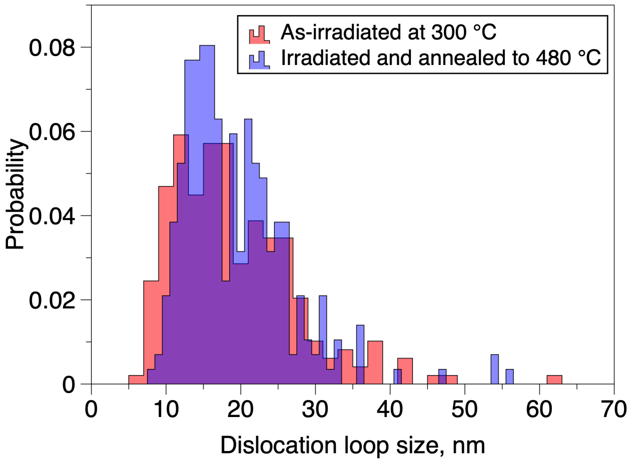

Figure S12: The size distribution of dislocation loops in the as-irradiated sample is similar to that for the sample annealed to 480°C. Dislocation loop size is defined as the Feret diameter measured using ImageJ, and 245 and 287 loops were measured for each sample, respectively. The mean loop diameter is 19 nm for both samples.

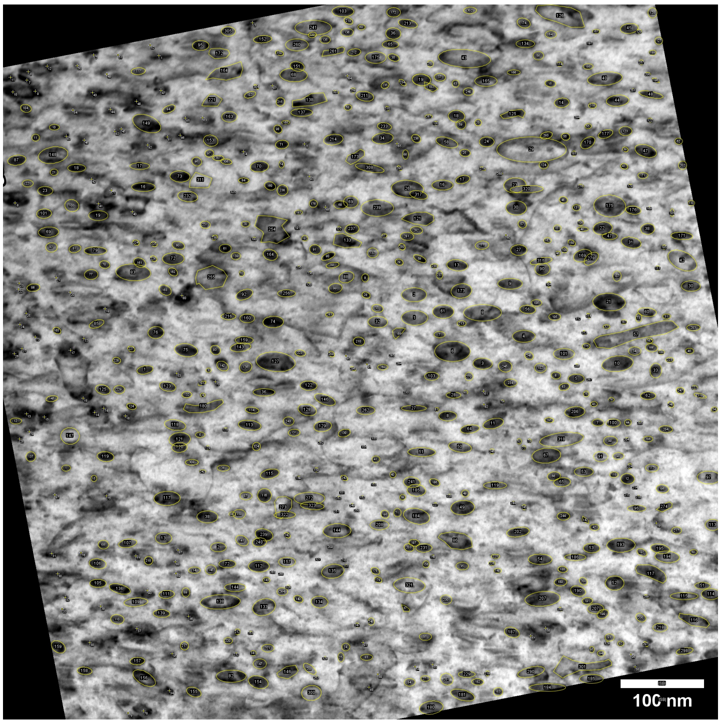

(a) Image analysis of the sample as-irradiated at 300°C.

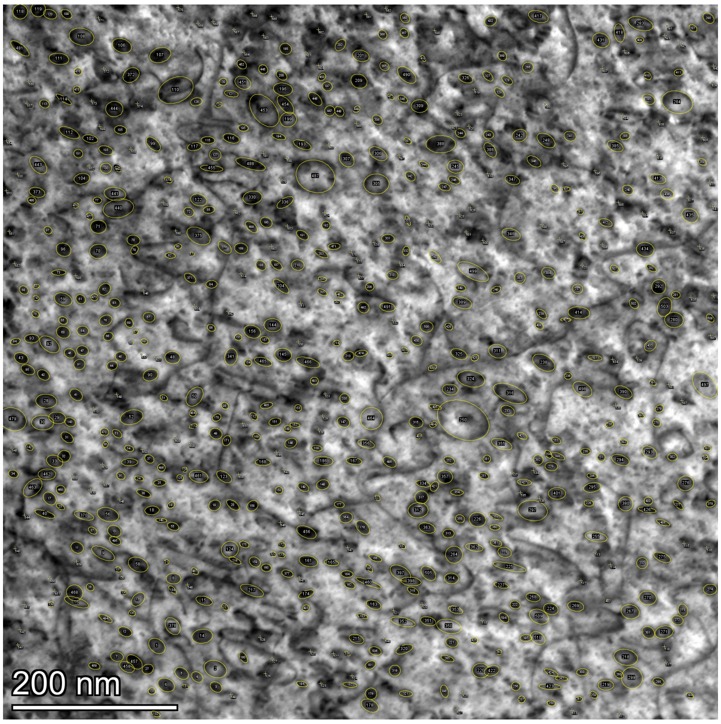

(b) Image analysis of the sample irradiated and annealed to 480°C.

Figure S13: ImageJ image analysis software was used to measure the dislocation loop size and number density. The number density was determined by counting the number of loops within the image and dividing by the sample volume. The as-irradiated sample contains a dislocation loop number density of $(4.0{\pm}0.7){\times}10^{21}$ m^{-3} and the irradiated and annealed to 480°C sample contains $(4.0{\pm}0.8){\times}10^{21}$ m^{-3}.

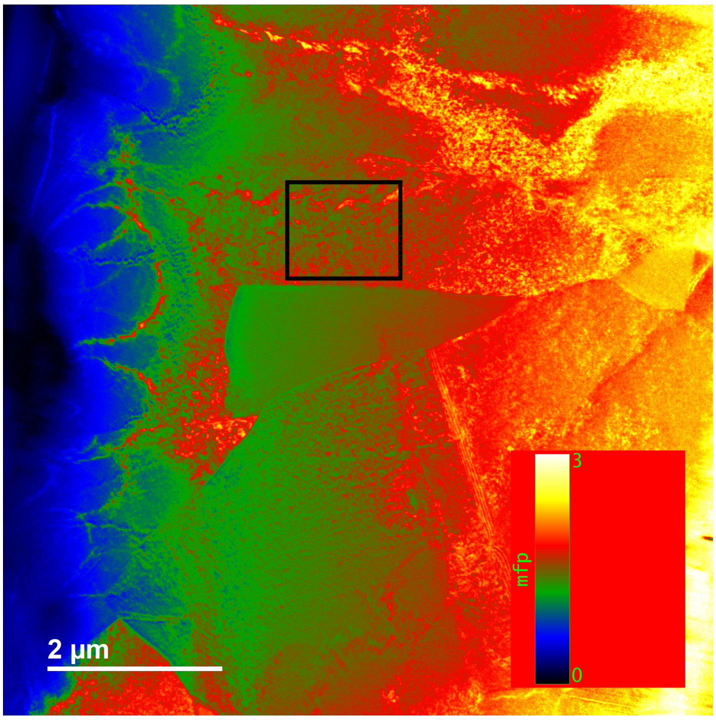

(a) EFTEM of sample as-irradiated at 300°C.

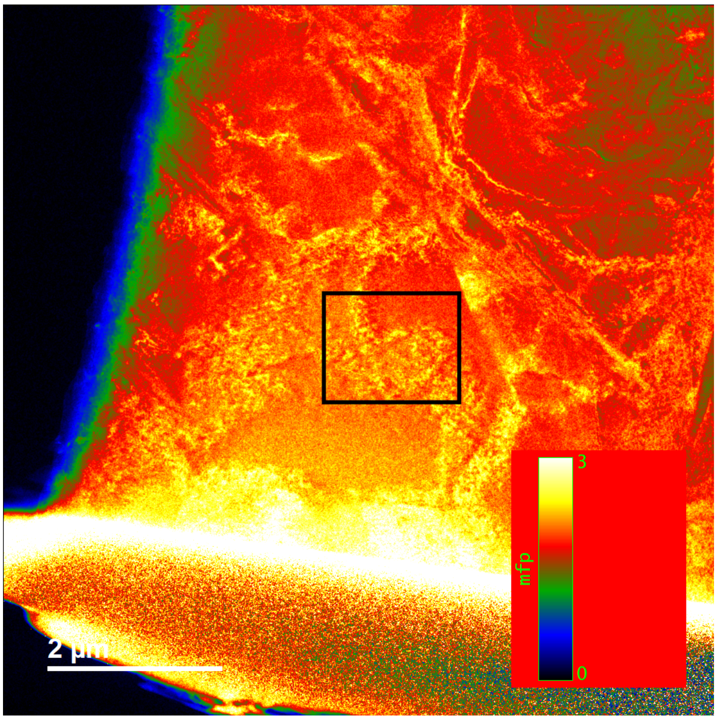

(b) EFTEM of sample irradiated and annealed to 480°C.

Figure S14: The thickness of the lamellae was determined using energy-filtered TEM (EFTEM). The black boxes show the locations of the images in figure S13, and their dimensions are (1029 nm)^2 and (858 nm)^2, respectively. The color scale indicates the inelastic mean free path of 200 keV electrons.

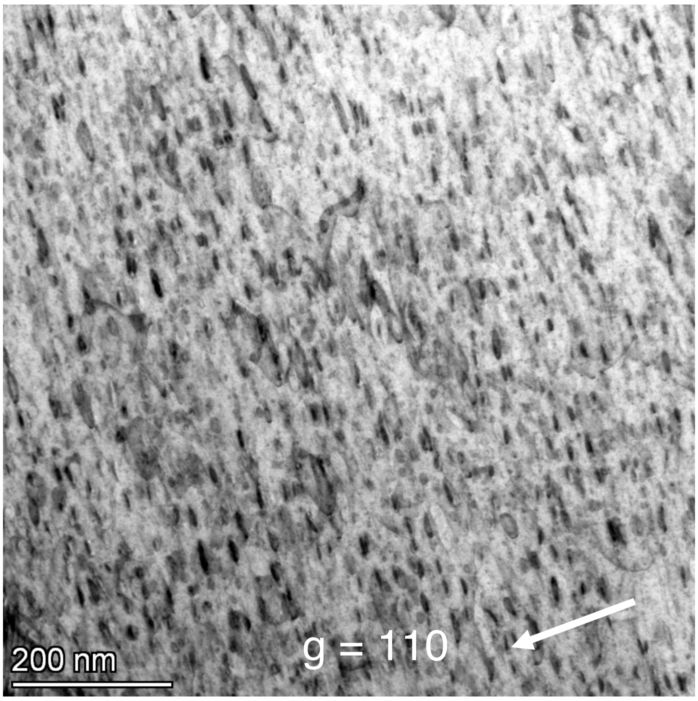

(a)

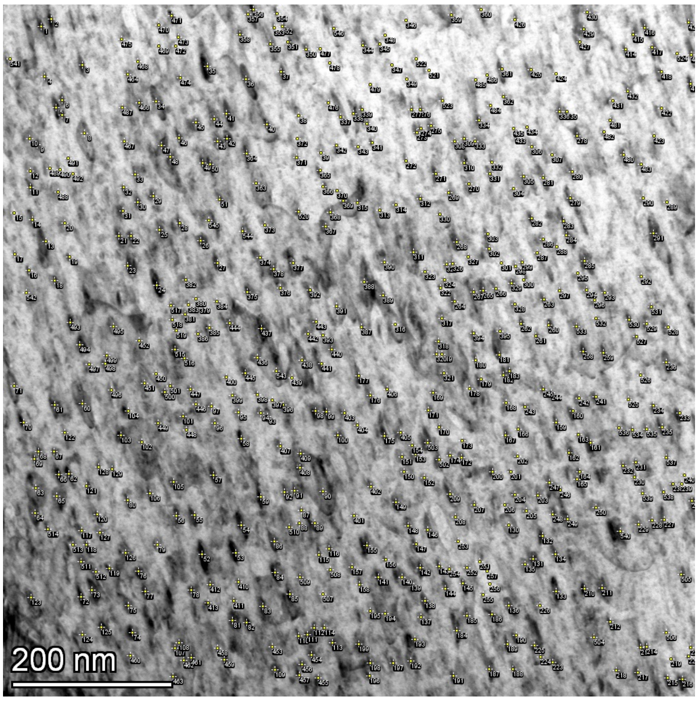

(b)

Figure S15: Imaging with different conditions returns the same number density of loops. (a) STEM image taken with zone axis = $[\bar{1}10]$ and **g** = 110 in the two beam condition. (b) The same image with annotations showing the 546 loops that were counted. This results in a number density of $(4.4{\pm}0.8){\times}10^{21}$ m^{-3} which is identical (within uncertainty) to the number density obtained earlier under different imaging conditions.

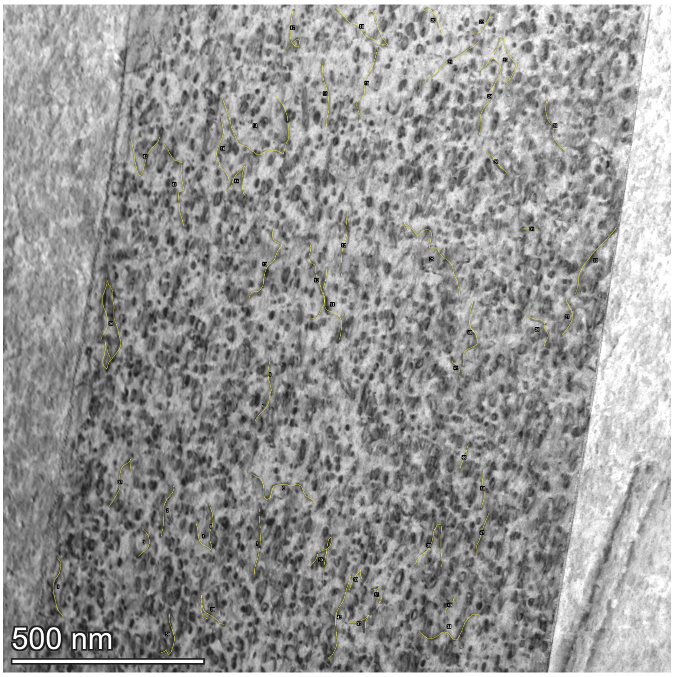

(a) As-irradiated sample. STEM image taken with a zone axis of [100] and **g** = 011.

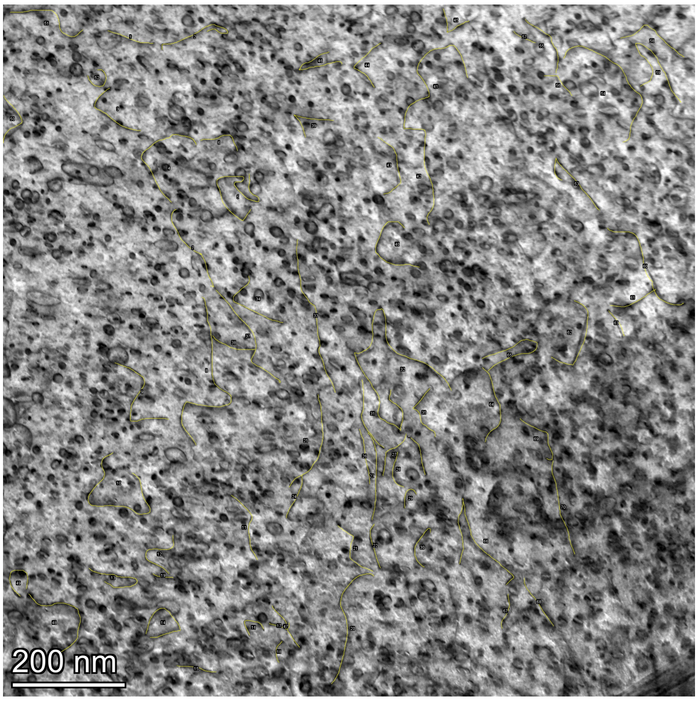

(b) Sample irradiated and annealed to 480° C.

Figure S16: Linear dislocations have an order of magnitude lower areal density than loops. Network dislocation density in the as-irradiated sample is $(2.4\pm0.5)\times10^{13}\,\mathrm{m}^{-2}$ and $(3.2\pm0.6)\times10^{13}\,\mathrm{m}^{-2}$ in the sample irradiated and annealed to 480°C.

REFERENCES AND NOTES

1. B. D. Wirth, How does radiation damage materials? *Science* **318**, 923–924 (2007).

2. X. Yi, A. Sand, D. Mason, M. Kirk, S. Roberts, K. Nordlund, S. Dudarev, Direct observation of size scaling and elastic interaction between nano-scale defects in collision cascades. *EPL* **110**, 36001 (2015).

3. Z. Zhou, S. L. Dudarev, M. L. Jenkins, A. P. Sutton, M. A. Kirk, Diffraction imaging and diffuse scattering by small dislocation loops. *J. Nucl. Mater.* **367-370**, 305–310 (2007).

4. E. Meslin, M. Lambrecht, M. Hernández-Mayoral, F. Bergner, L. Malerba, P. Pareige, B. Radiguet, A. Barbu, D. Gómez-Briceño, A. Ulbricht, A. Almazouzi, Characterization of neutron-irradiated ferritic model alloys and a RPV steel from combined APT, SANS, TEM and PAS analyses. *J. Nucl. Mater.* **406**, 73–83 (2010).

5. A. Reza, H. Yu, K. Mizohata, F. Hofmann, Thermal diffusivity degradation and point defect density in self-ion implanted tungsten. *Acta Mater.* **193**, 270–279 (2020).

6. M. Eldrup, B. Singh, Studies of defects and defect agglomerates by positron annihilation spectroscopy. *J. Nucl. Mater.* **251**, 132–138 (1997).

7. J. A. Horak, T. H. Blewitt, Fast- and thermal-neutron irradiation and annealing of Cu, Ni, Fe, Ti, and Pd. *Nucl. Technol.* **27**, 416–438 (1975).

8. G. Imbalzano, "First principle calculations of the residual resistivity of defects in metals," thesis, KTH Royal Institute of Technology, Stockholm Sweden (2015).

9. H. E. Kissinger, Reaction kinetics in differential thermal analysis. *Anal. Chem.* **29**, 1702–1706 (1957).

10. E. P. Wigner, Theoretical physics in the metallurgical laboratory of Chicago. *J. Appl. Phys.* **17**, 857–863 (1946).

11. T. Iwata, Fine structure of Wigner energy release spectrum in neutron irradiated graphite. *J. Nucl. Mater.* **133-134**, 361–364 (1985).

12. R. H. Telling, C. P. Ewels, A. A. El-Barbary, M. I. Heggie, Wigner defects bridge the graphite gap. *Nat. Mater.* **2**, 333–337 (2003).

13. S. Roorda, W. C. Sinke, J. M. Poate, D. C. Jacobson, S. Dierker, B. S. Dennis, D. J. Eaglesham, F. Spaepen, P. Fuoss, Structural relaxation and defect annihilation in pure amorphous silicon. *Phys. Rev. B* **44**, 3702–3725 (1991).

14. L. L. Snead, Y. Katoh, T. Koyanagi, K. Terrani, Stored energy release in neutron irradiated silicon carbide. *J. Nucl. Mater.* **514**, 181–188 (2019).

15. R. Losehand, F. Rau, H. Wenzl, Stored energy and electrical resistivity of Frenkel defects in copper. *Radiat. Eff.* **2**, 69–74 (1969).

16. K. Isebeck, F. Rau, W. Schilling, K. Sonnenberg, P. Tischer, H. Wenzl, Stored energy, volume, and resistivity change in neutron irradiated aluminium. *Phys. Status Solidi B Basic Res.* **17**, 259–268 (1966).

17. J. C. Nicoud, E. Bonjour, D. Schumacher, J. Delaplace, Restauration dans le stade i du beryllium irradie par des neutrons et par des electrons. *Phys. Lett. A* **26**, 228–229 (1968).

18. J. Delaplace, J. Hillairet, J. C. Nicoud, D. Schumacher, G. Vogl, Low temperature neutron radiation damage and recovery in magnesium. *Phys. Status Solidi B Basic Res.* **30**, 119–126 (1968).

19. M.-J. Caturla, M. Wall, E. Alonso, T. D. de la Rubia, T. Felter, M. Fluss, Heavy ion irradiation and annealing of lead: Atomistic simulations and experimental validation. *J. Nucl. Mater.* **276**, 186–193 (2000).

20. W. Schilling, Radiation induced damage in metals. *J. Nucl. Mater.* **72**, 1–4 (1978).

21. M. Griffiths, D. Faulkner, R. C. Styles, Neutron damage in α-titanium. *J. Nucl. Mater.* **119**, 189–207 (1983).

22. M. L. Jenkins, Characterisation of radiation-damage microstructures by TEM. *J. Nucl. Mater.* **216**, 124–156 (1994).

23. Y. Higashiguchi, H. Kayano, S. Yajima, Relation between microstructure and recovery behavior in fast-neutron irradiated titanium. *J. Nucl. Sci. Technol.* **13**, 454–458 (1976).

24. M. Hasegawa, S. Kioke, M. Hirabayashi, Y. Higashiguchi, E. Kuramoto, K. Kitajima, A positron annihilation study of neutron irradiated titanium, in *Point Defects and Defect Interactions in Metals* (J-Global, 1982).

25. K. Hajizadeh, S. G. Alamdari, B. Eghbali, Stored energy and recrystallization kinetics of ultrafine grained titanium processed by severe plastic deformation. *Phys. B Condens. Matter* **417**, 33–38 (2013).

26. A. J. Prabha, S. Raju, H. Tripathy, M. Vijayalakshmi, I. Johnson, Kinetics of thermally activated lattice softening in titanium: A dynamic calorimetry study. *Trans. Indian Inst. Met.* **66**, 415–418 (2013).

27. M. Topping, T. Ungár, C. P. Race, A. Harte, A. Garner, F. Baxter, S. Dumbill, P. Frankel, M. Preuss, Investigating the thermal stability of irradiation-induced damage in a zirconium alloy with novel in situ techniques. *Acta Mater.* **145**, 255–263 (2018).

28. P. M. Derlet, S. L. Dudarev, Microscopic structure of a heavily irradiated material. *Phys. Rev. Mater.* **4**, 023605 (2020).

29. B. Mastel, H. E. Kissinger, J. J. Laidler, T. K. Bierlein, Dislocation channeling in neutron-irradiated molybdenum. *J. Appl. Phys.* **34**, 3637–3638 (1963).

30. T. D. de la Rubia, H. M. Zbib, T. A. Khraishi, B. D. Wirth, M. Victoria, M. J. Caturla, Multiscale modelling of plastic flow localization in irradiated materials. *Nature* **406**, 871–874 (2000).

31. C.-C. Fu, J. D. Torre, F. Willaime, J.-L. Bocquet, A. Barbu, Multiscale modelling of defect kinetics in irradiated iron. *Nat. Mater.* **4**, 68–74 (2004).

32. V. N. Bykov, G. A. Birzhevoi, M. A. Zakharova, V. A. Solov’ev, Nature and thermal stability of radiation defects in single-crystal tungsten. *Sov. At. Energy* **33**, 930–935 (1972).

33. T. H. Blewitt, S. T. Sekula, J. Diehl, Energy release in reactor-irradiated copper. II. 600° to 700°K release. *Phys. Rev.* **122**, 53–57 (1961).

34. C. A. Dennett, B. R. Dacus, C. M. Barr, T. Clark, H. Bei, Y. Zhang, M. P. Short, K. Hattar, The dynamic evolution of swelling in nickel concentrated solid solution alloys through in situ property monitoring. *Appl. Mater. Today* **25**, 101187 (2021).

35. M. J. Norgett, M. T. Robinson, I. M. Torrens, A proposed method of calculating displacement dose rates. *Nucl. Eng. Des.* **33**, 50–54 (1975).

36. G. Was, *Fundamentals of Radiation Materials Science* (Springer, ed. 2, 2017).

37. A. Cezairliyan, F. Righini, Thermodynamic studies of the α → β phase transformation in zirconium using a subsecond pulse heating technique. *J. Res. Natl. Bur. Stand. A Phys. Chem.* **79A**, 81–84 (1975).

38. T. Malis, S. C. Cheng, R. F. Egerton, EELS log-ratio technique for specimen-thickness measurement in the TEM. *J. Electron Microsc. Tech.* **8**, 193–200 (1988).

39. H. Shinotsuka, S. Tanuma, C. J. Powell, D. R. Penn, Calculations of electron inelastic mean free paths. X. data for 41 elemental solids over the 50 eV to 200 keV range with the relativistic full Penn algorithm. *Surf. Interf. Anal.* **47**, 871–888 (2015).

40. J. P. Hirth, J. Lothe, *Theory of Dislocations* (MMl: Krieger Publishing Group, ed. 2, 1982).

41. S.-M. Liu, I. J. Beyerlein, W.-Z. Han, Two-dimensional vacancy platelets as precursors for basal dislocation loops in hexagonal zirconium. *Nat. Commun.* **11**, 5766 (2020).

42. K. Nordlund, M. Ghaly, R. S. Averback, M. Caturla, T. Diaz de la Rubia, J. Tarus, Defect production in collision cascades in elemental semiconductors and fcc metals. *Phys. Rev. B* **57**, 7556–7570 (1998).

43. K. Nordlund, Molecular dynamics simulation of ion ranges in the 1-100 keV energy range. *Comput. Mater. Sci.* **3**, 448–456 (1995).

44. G. J. Ackland, Theoretical study of titanium surfaces and defects with a new many-body potential. *Philos. Mag. A* **66**, 917–932 (1992).

45. G. Ackland, Ti potential with close-range repulsion; https://www.ctcms.nist.gov/potentials/entry/1992--Ackland-G-J--Ti/1992--Ackland-G-J--Ti--LAMMPS--ipr2.html [accessed 12 August 2021].

46. S. Plimpton, Fast parallel algorithms for short-range molecular dynamics. *J. Comput. Phys.* **117**, 1–19 (1995).

47. A. Stukowski, Visualization and analysis of atomistic simulation data with OVITO-the open visualization tool. *Model. Simul. Mat. Sci. Eng.* **18**, 015012 (2010).

48. A. Stukowski, V. V. Bulatov, A. Arsenlis, Automated identification and indexing of dislocations in crystal interfaces. *Model. Simul. Mat. Sci. Eng.* **20**, 085007 (2012).

49. M. J. Caturla, N. Soneda, E. Alonso, B. D. Wirth, T. D. de la Rubia, J. M. Perlado, Comparative study of radiation damage accumulation in Cu and Fe. *J. Nucl. Mater.* **276**, 13–21 (2000).

50. B. D. Wirth, X. Hu, A. Kohnert, D. Xu, Modeling defect cluster evolution in irradiated structural materials: Focus on comparing to high-resolution experimental characterization studies. *J. Mater. Res.* **30**, 1440–1455 (2015).

51. C. Liu, L. He, Y. Zhai, B. Tyburska-Püschel, P. M. Voyles, K. Sridharan, D. Morgan, I. Szlufarska, Evolution of small defect clusters in ion-irradiated 3C-SIC: Combined cluster dynamics modeling and experimental study. *Acta Mater.* **125**, 377–389 (2017).

52. H. Jiang, L. He, D. Morgan, P. M. Voyles, I. Szlufarska, Radiation-induced mobility of small defect clusters in covalent materials. *Phys. Rev. B* **94**, 024107 (2016).

53. Y. N. Osetsky, D. J. Bacon, A. Serra, B. N. Singh, S. I. Golubov, Stability and mobility of defect clusters and dislocation loops in metals. *J. Nucl. Mater.* **276**, 65–77 (2000).

54. K. Song, S. Das, A. Reza, N. W. Phillips, R. Xu, H. Yu, K. Mizohata, D. E. J. Armstrong, F. Hofmann, Characterising ion-irradiated FeCr: Hardness, thermal diffusivity and lattice strain. *Acta Mater.* **201**, 535–546 (2020).

55. O. J. Weiß, E. Gaganidze, J. Aktaa, Quantitative characterization of microstructural defects in up to 32dpa neutron irradiated Eurofer97. *J. Nucl. Mater.* **426**, 52–58 (2012).

56. C. Kittel, *Introduction to Solid State Physics* (Wiley, ed. 8, 2004).

57. J. W. Martin, The electrical resistivity of some lattice defects in FCC metals observed in radiation damage experiments. *J. Phys. F* **2**, 842–853 (1972).

58. N. Hiroshi, Defects in Metals, in *Physical Metallurgy* (Elsevier, 2015), pp. 604–605.

59. S. Takaki, J. Fuss, H. Kuglers, U. Dedek, H. Schultz, The resistivity recovery of high purity and carbon doped iron following low temperature electron irradiation. *Radiat. Eff.* **79**, 87–122 (1983).

60. K. Nordlund, S. J. Zinkle, A. E. Sand, F. Granberg, R. S. Averback, R. E. Stoller, T. Suzudo, L. Malerba, F. Banhart, W. J. Weber, F. Willaime, S. L. Dudarev, D. Simeone, Primary radiation damage: A review of current understanding and models. *J. Nucl. Mater.* **512**, 450–479 (2018).

61. K. S. Pedchenko, V. S. Karasev, Stored energy in neutron-bombarded metals. *Sov. Mater. Sci.* **4**, 332–336 (1971).

62. G. Kinchin, M. Thompson, Irradiation damage and recovery in molybdenum and tungsten. *J. Nucl. Energy (1954)* **6**, 275–284 (1958).

63. D. Toktogulova, M. Gusev, O. Maksimkin, F. Garner, Influence of neutron irradiation on energy accumulation and dissipation during plastic flow and hardening of metallic polycrystals. *J. ASTM Int.* **7**, (2010).

64. B.-H. Lee, J.-S. Cheon, Y.-H. Koo, J.-Y. Oh, J.-S. Yim, D.-S. Sohn, M. Baryshnikov, A. Gaiduchenko, Measurement of the specific heat of Zr-40 wt% U metallic fuel. *J. Nucl. Mater.* **360**, 315–320 (2007).

65. S. M. Ennaceur, A. Migliori, Toward an understanding of aging in plutonium from direct measurements of stored energy. *Philos. Mag. Lett.* **98**, 502–510 (2018).

66. L. K. Béland and N. Mousseau, Long-time relaxation of ion-bombarded silicon studied with the kinetic activation-relaxation technique: Microscopic description of slow aging in a disordered system. *Phys. Rev. B* **88**, 214201 (2013).

67. J. L. Brimhall, G. L. Kulcinski, H. E. Kissinger, B. Mastel, Microstructural analysis of neutron irradiated titanium and rhenium. *Radiat. Eff.* **9**, 273–278 (1971).

68. A. Jostsons, R. G. Blake, P. M. Kelly, Characterization of dislocation loops in neutron-irradiated titanium. *Philos. Mag.* **41**, 903–916 (1980).

69. M. Griffiths, C. D. Cann, R. C. Styles, Neutron irradiation damage in 64% cold-worked titanium. *J. Nucl. Mater.* **149**, 200–211 (1987).

70. R. H. Jones, L. A. Charlot, Microstructure of irradiated Ti-70a and Ti-6Al-4V. *J. Nucl. Mater.* **91**, 329–335 (1980).

71. R. J. Contieri, M. Zanotello, R. Caram, Recrystallization and grain growth in highly cold worked CP-titanium. *Mater. Sci. Eng. A* **527**, 3994–4000 (2010).

72. E. Kaschnitz, P. Reiter, Heat capacity of titanium in the temperature range 1500 to 1900K measured by a millisecond pulse-heating technique. *J. Therm. Anal. Calorim.* **64**, 351–356 (2001).

73. T. Ślęzak, P. Koniorczyk, J. Zmywaczyk, DSC investigations of titanium grade 1 in a wide temperature range. *AIP Conf. Proc.* **2170**, 020020 (2019).

74. B. Kombaiah, K. L. Murty, High temperature creep and deformation microstructures in recrystallized Zircaloy-4. *Philos. Mag.* **95**, 1656–1679 (2015).

75. E. S. Fisher, C. J. Renken, Single-crystal elastic moduli and the hcp → bcc transformation in Ti, Zr, and Hf. *Phys. Rev.* **135**, A482–A494 (1964).

76. R. Boyer, G. Welsch, E. W. Collings, *Materials Properties Handbook: Titanium Alloys* (ASM International, 1994).

77. M. M. Savin, V. M. Chernov, A. M. Strokova, Energy factor of dislocations in hexagonal crystals. *Phys. Status Solidi A* **35**, 747–754 (1976).
